# Hematological Toxicities in the Modern Era of Melanoma Therapy

**DOI:** 10.3390/jcm15166296

**Published:** 2026-08-14

**Authors:** Rodica Anghel, Ana-Maria Zamfirescu-Deryder, Vlad-Luca Moga, Antonia-Ruxandra Folea, Radu-Valeriu Toma, Andreea-Iren Șerban, Liviu Bîlteanu

**Affiliations:** 1 Faculty of General Medicine, Carol Davila University of Medicine and Pharmacy, 8 Eroii Sanitari Street, 050474 Bucharest, Romania; rodica.anghel@umfcd.ro (R.A.); vlad-luca.moga@drd.umfcd.ro (V.-L.M.); antonia-ruxandra.folea@drd.umfcd.ro (A.-R.F.); radu.toma@umfcd.ro (R.-V.T.); liviu.bilteanu@umfcd.ro (L.B.); 2 Oncological Institute “Alexandru Trestioreanu” Bucharest, 252 Soseaua Fundeni, 022328 Bucharest, Romania; 3 Faculty of Biology, University of Bucharest, 91-95 Splaiul Independentei, 050095 Bucharest, Romania; andreea-iren.serban@fmvb.usamv.ro; 4 Department of Preclinical Sciences, Faculty of Veterinary Medicine, University of Agronomic Sciences and Veterinary Medicine, 105 Splaiul Independentei, 050097 Bucharest, Romania; 5 Laboratory for Molecular Nanotechnologies, National Institute for Research and Development in Microtechnologies—IMT Bucharest, 126A, Erou Iancu Nicolae Street, 077190 Voluntari, Romania

**Keywords:** melanoma, immunotherapy, immune checkpoint inhibitors, hematological toxicities, immune-related adverse events (irAEs), targeted therapy, cytopenia, autoimmunity

## Abstract

**Background/Objectives:** The advent of immune checkpoint inhibitors (ICIs) and targeted therapies has revolutionized advanced melanoma treatment but introduced unique immune-related adverse events (irAEs). Hematological irAEs (Hem-irAEs) are rare but carry disproportionately high morbidity and mortality. This review systematically synthesizes current literature to comprehensively understand the incidence, pathophysiology, clinical presentation, and management of Hem-irAEs in modern melanoma therapy. **Methods:** A comprehensive Web of Science literature search (January 2015 to January 2026) identified studies reporting hematological adverse events associated with melanoma immunotherapy and targeted therapies. After screening 2274 records, 130 relevant studies were included for quantitative data extraction, focusing on incidence rates and toxicity grading. **Results:** Hem-irAEs occur infrequently (under 4% overall incidence for ICIs) but possess staggering mortality rates between 12% and 15.5%. The most common manifestations are immune thrombocytopenia (ITP), autoimmune hemolytic anemia, and neutropenia. Combination regimens significantly amplify toxicity frequency and severity. Diagnosis requires meticulous baseline monitoring and bone marrow biopsies to differentiate peripheral destruction from central marrow failure. First-line management mandates ICI discontinuation and high-dose corticosteroids, utilizing targeted second-line immunosuppressants for refractory syndromes. **Conclusions:** Hem-irAEs embody a profound clinical paradox: while mild toxicities often herald a robust anti-tumor response, severe hematological events drastically increase non-cancer mortality, negating these oncological benefits. Navigating this “double-edged sword” demands a paradigm shift toward proactive risk stratification. Integrating predictive biomarkers including baseline autoantibodies, Human Leukocyte Antigens (HLA) profiling, and systemic inflammatory indices is crucial to identify vulnerable populations before treatment. Optimizing outcomes requires highly personalized vigilance to balance the life-saving efficacy of immunotherapy against the catastrophic threat of hematopoietic failure.

## 1. Introduction

### 1.1. The Revolution in Melanoma Therapeutics

Historically, advanced melanoma was a highly aggressive tumor characterized by a very poor prognosis. Prior to the advent of modern treatments, systemic therapy primarily relied on traditional cytotoxic chemotherapy (such as dacarbazine) or high-dose interleukin-2 (IL-2), which yielded low response rates of only 10% to 20% and generally failed to significantly improve long-term overall survival (OS) [1,2,3,4].

Over the last decade, the therapeutic landscape has undergone a dramatic paradigm shift, bringing about novel therapies that have significantly prolonged both progression-free survival (PFS) and OS [5,6,7]. This revolution in modern melanoma therapy is built upon two major treatment pillars: targeted therapies (specifically BRAF and MEK inhibitors like vemurafenib, dabrafenib, and trametinib) and immune checkpoint inhibitors (ICIs) [2,8,9,10].

Unlike conventional chemotherapy, ICIs do not directly attack the tumor but instead counteract tumor-mediated immune suppression. They utilize monoclonal antibodies to target and block key co-inhibitory receptors, specifically cytotoxic T-lymphocyte-associated protein 4 (CTLA-4, e.g., ipilimumab) and programmed cell death protein 1 (PD-1, e.g., nivolumab, pembrolizumab) or its ligand (PD-L1) [11,12]. By inhibiting these pathways and removing the physiological “brakes” on the immune system, ICIs unleash a potent, sustained, T-cell-mediated anti-tumor immune response** capable of recognizing and destroying malignant cells [13,14]. Despite increasing the risk for manageable hematological side effects, incorporating VEGF blockade into combination regimens has shown considerable benefit in prolonging OS for patients with unresectable stage III or IV melanoma [9].

### 1.2. Emergence and Paradigm Shift in Treatment Toxicities

The introduction of modern immunotherapy has brought a paradigm shift not only in cancer survival but also in the profile of treatment-related toxicities. While conventional cytotoxic chemotherapy is well-known for causing predictable, dose-dependent side effects such as myelosuppression and cytopenias due to direct bone marrow toxicity [15], ICIs produce a completely distinct and less predictable spectrum of toxicities [16,17]. These unique toxicities, recognized as immune-related adverse events (irAEs), closely mimic autoimmune and inflammatory conditions rather than typical cytotoxic drug reactions [14,18].

The emergence of irAEs is an unavoidable consequence of the unique mechanism of action of ICIs. By removing inhibitory signals like CTLA-4 and PD-1, ICIs induce unregulated systemic immune activation. While this effectively targets malignant cells, it simultaneously risks collateral autoimmune-like inflammatory damage across healthy host tissues [12,19,20,21,22,23].

Importantly, the therapeutic landscape has increasingly moved toward dual checkpoint blockade to overcome tumor resistance and improve clinical outcomes. However, combining immunotherapies, most notably the anti-CTLA-4 agent ipilimumab plus the anti-PD-1 agent nivolumab, acts as a double-edged sword. While this combination significantly increases antitumor efficacy and OS in metastatic melanoma, it concurrently amplifies the frequency and severity of irAEs [24,25]. Clinical data indicate that severe, high-grade (Grade 3 or 4) irAEs occur in upwards of 55% to 59% of patients receiving combination therapy, a rate that is markedly higher and leads to more frequent treatment discontinuation than either agent administered as monotherapy [13,14,26]. As an alternative to systemic checkpoint blockade, novel targeted vaccination strategies such as dendritic cell-targeted liposomal vaccines are actively being evaluated to stimulate anti-melanoma immunity with a much more favorable toxicity profile [6].

In general, severe adverse events associated with anti-PD-1 and anti-PD-L1 therapies appear to be less frequent compared with anti-CTLA-4 therapies, although they still require strict monitoring [27]. In patients with advanced melanoma whose disease has progressed on ipilimumab, PD-1 blockade has demonstrated superior PFS and lower rates of severe hematologic toxicity compared with investigator-choice chemotherapy [10].

### 1.3. The Spectrum of irAEs: From Common Manifestations to Rare Hematological Events

The systemic immune activation induced by ICIs can affect virtually any organ, leading to a wide array of irAEs. The most frequently observed irAEs primarily involve the skin (manifesting as maculopapular rash, pruritus, and vitiligo), the gastrointestinal tract (presenting as diarrhea and colitis), the endocrine glands (frequently causing thyroiditis and hypophysitis), and the liver (resulting in hepatitis) [12,14]. These common toxicities are typically mild to moderate in severity and have well-established management algorithms [28,29]. Given that the pharmacodynamic and immunological effects of monoclonal antibodies can persist extensively, irAEs often demand prolonged vigilance and careful management well beyond the administration period [20].

However, beyond these common presentations lies a rare but clinically severe subset of irAEs that carries a much higher risk of morbidity and rapid clinical deterioration. This subset includes cardiovascular toxicities (such as fatal myocarditis), neurological complications (such as encephalitis and Guillain-Barré syndrome), and hematological toxicities [18,27,30,31,32].

Hematological irAEs (Hem-irAEs) are defined as a diverse and potentially life-threatening group of immune-mediated blood disorders that arise from the loss of immunological self-tolerance [33,34]. While their overall incidence is low, they require immediate recognition due to their fulminant nature. The specific conditions seen in clinical practice encompass a broad spectrum of cytopenias and systemic syndromes. Most notably, these include autoimmune hemolytic anemia (AIHA), immune thrombocytopenia (ITP), profound neutropenia, and severe pancytopenia [27,35,36,37,38,39,40]. Furthermore, clinicians may encounter exceptionally rare and catastrophic manifestations, such as acquired amegakaryocytic thrombocytopenia (AAT), pure red cell aplasia (PRCA), hemophagocytic lymphohistiocytosis (HLH), and thrombotic thrombocytopenic purpura (TTP) [25,41,42,43].

### 1.4. The Clinical Significance and Mortality Burden of Hematological Toxicities

Although ICIs are generally better tolerated than traditional cytotoxic chemotherapy, Hem-irAEs represent a rare but exceptionally dangerous subset of toxicities. Although they occur much less frequently than other irAEs, hematological toxicities are associated with a disproportionately high morbidity and mortality burden, making early clinical recognition critical [35,41].

Despite their relatively low frequency, these specific events are associated with disproportionately high morbidity and mortality. Severe bone marrow failure syndromes, in particular, represent acute oncological emergencies, occasionally driving fatal outcomes due to infectious or hemorrhagic complications [44,45,46]. This high risk of rapid clinical deterioration underscores the critical importance of prompt identification and aggressive supportive management. Furthermore, conditions like HLH carry a remarkably high mortality burden despite immunosuppressive treatment, and profound Grade 4 neutropenia frequently becomes fatal when complicated by overwhelming bacterial or fungal sepsis [33,35]. Importantly, the administration of ICIs also heightens the risk for venous and arterial thromboembolism, which serves as an independent predictor of diminished OS in melanoma [47]. Although relatively rare, severe ITP induced solely by CTLA-4 blockade can follow a fulminant course and occasionally result in a fatal outcome if not aggressively managed [39]. Furthermore, severe lymphopenia at baseline or developing early during treatment has been identified as a powerful independent predictor of toxicity and early mortality after cytotoxic therapies [48].

Compounding their lethal potential, significant delays in the diagnosis of Hem-irAEs are common. The early hematologic manifestations can be incredibly subtle and are frequently a diagnosis of exclusion. These abnormalities often mimic cancer progression, paraneoplastic syndromes, or opportunistic infectious complications, and can easily overlap with the expected bone marrow-suppressive side effects of other concurrent medications [15,47,48,49,50,51,52,53,54]. In systemic events like HLH, the heterogeneous presentation frequently overlaps with confounding factors like the underlying malignancy or sepsis, further complicating and delaying timely clinical recognition and intervention [55].

### 1.5. Rationale and Purpose of the Review

As the indications for ICIs continue to rapidly expand in the treatment of advanced melanoma and other solid tumors, the absolute number of exposed patients is growing exponentially [18,34]. Consequently, encountering these rare but potentially fatal Hem-irAEs will become increasingly common in routine clinical practice, necessitating heightened clinician vigilance and awareness [36,56].

Currently, the available evidence regarding Hem-irAEs remains scarce and highly fragmented, consisting predominantly of isolated case reports, small case series, and retrospective pharmacovigilance studies [45,56,57]. Therefore, the primary objective of this review is to systematically synthesize this scattered current literature to establish a comprehensive understanding of the incidence, underlying pathophysiology, clinical presentation, and multidisciplinary management algorithms for hematological toxicities in the modern era of melanoma therapy [34,35].

## 2. Materials and Methods

### 2.1. Literature Search

A comprehensive literature search was performed in February 2026 using Web of Science databases, covering studies published between January 2015 and January 2026. The search formula employed the following query to filter relevant articles and abstracts; the search was conducted within the title and abstract fields:

TS = (melanoma OR “cutaneous melanoma” OR “metastatic melanoma”) AND TS = ( immunotherapy OR “immune checkpoint inhibitor*” OR “checkpoint inhibitor*” OR “anti-PD-1” OR “anti-PD-L1” OR “anti-CTLA-4” OR “targeted therapy” OR “BRAF inhibitor*” OR “MEK inhibitor*” OR chemotherapy) AND TS = (“hematologic adverse event*” OR “hematological adverse event*” OR “hematologic toxicit*” OR “hematological toxicit*” OR cytopenia* OR anemia OR thrombocytopenia OR neutropenia OR pancytopenia OR “bone marrow” OR “aplastic anemia” OR hemophagocytic OR hemophagocytosis OR “immune-mediated” OR autoimmune).

**Figure 1:**
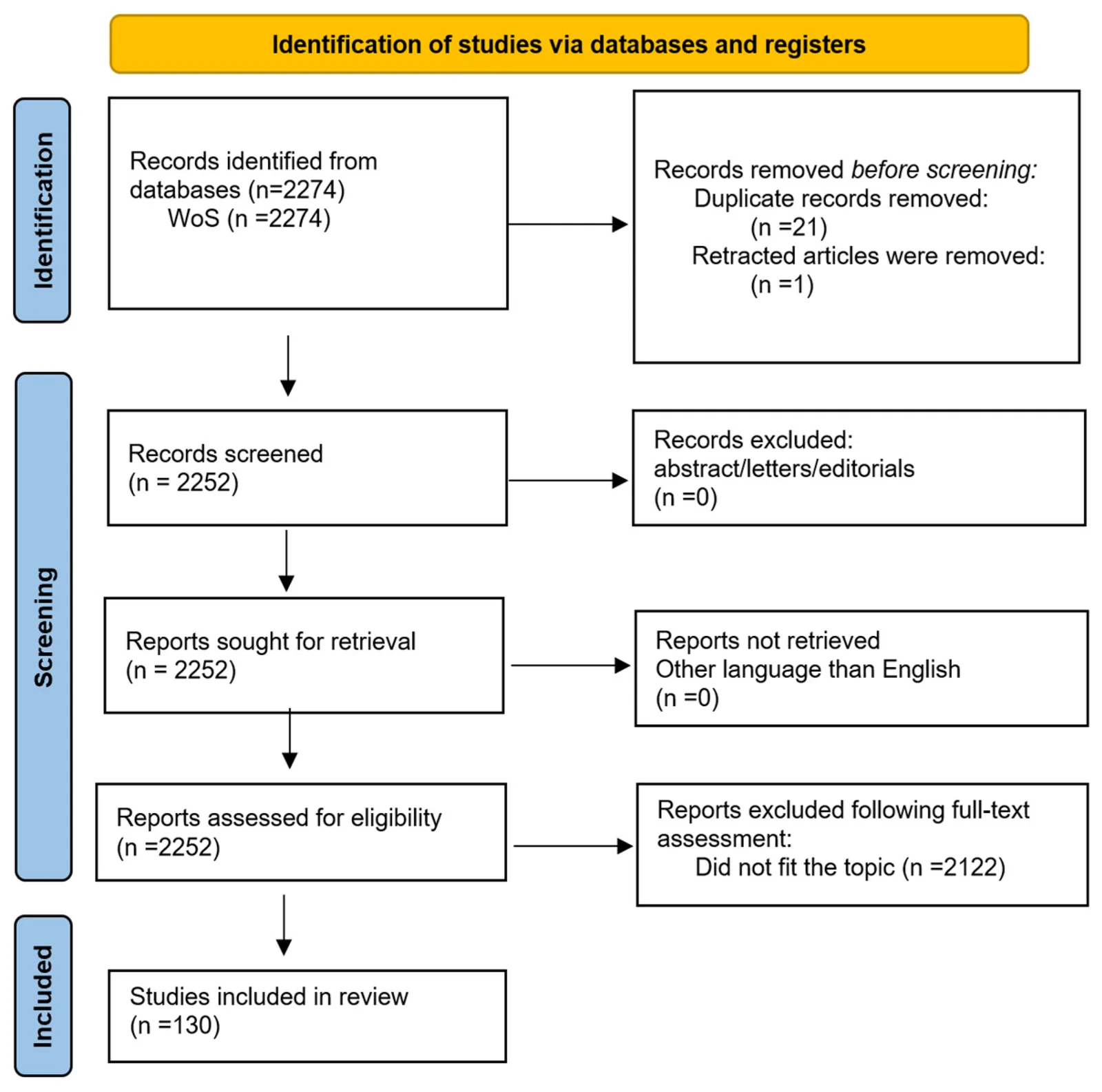
Flow-chart showing the selection processes.

This search strategy yielded a very large number of records; however, from this extensive body of literature, we selected only those studies that reported exclusively hematological adverse events (anemia, thrombocytopenia, lymphopenia, neutropenia, and pancytopenia) associated with immunotherapy in solid tumors, with a particular focus on melanoma, ICIs, or targeted therapies. Quantitative data, including incidence rates and grading according to the Common Terminology Criteria for Adverse Events, were subsequently extracted, while studies that did not align with the focus of this analysis were excluded. The study selection process is summarized in the flowchart presented in Figure 1. Initially, 2274 records were identified across the three databases. After eliminating 21 duplicate records and 1 retracted article. Following further screening, 2122 records were excluded for not aligning with our objective to find extensive, reliable studies, leaving 130 records for inclusion. These consisted of 52 original articles, 31 reviews, 36 case reports, 9 meta-analyses, 1 guideline, and 1 consensus report.

### 2.2. Inclusion and Exclusion Criteria

To ensure a rigorous and relevant selection of the literature, we applied specific inclusion and exclusion criteria during the screening process.

Inclusion criteria for the papers were:

- Studies explicitly reporting hematological adverse events (e.g., anemia, thrombocytopenia, lymphopenia, neutropenia, pancytopenia, acquired amegakaryocytic thrombocytopenia, PRCA, hemophagocytic lymphohistiocytosis, and TTP).
- Studies evaluating patients with solid tumors, with a primary focus on melanoma, treated with ICIs or targeted therapies (BRAF/MEK inhibitors).
- Studies providing relevant qualitative clinical details or quantitative data, such as incidence rates and severity grading.
- Original research articles, comprehensive reviews, case reports, meta-analyses, clinical guidelines, and consensus reports.
- Articles published between January 2015 and January 2026.
- Full-text articles published in English.

On the other hand, the exclusion criteria were:

- Studies that did not focus on Hem-irAEs or were unrelated to immunotherapy/targeted therapy in melanoma and other solid tumors.
- Non-research articles with limited extractable data, including letters to the editor, short communications, and meeting abstracts.
- Duplicate records across the searched databases and retracted articles.

Finally, articles published in languages other than English were also excluded.

### 2.3. Quality Assessment

Given the heterogeneity of the included literature, which ranges from isolated case reports to large randomized controlled trials, study quality was assessed to ensure the reliability of the synthesized data. Because rare Hem-Iraes are predominantly documented in case reports, the quality of these records was assessed in terms of documentation, uniqueness, educational value, objectivity, and interpretation. Retrospective cohorts, single-arm trials, and large-scale real-world pharmacovigilance studies were assessed based on their endpoints, follow-up periods, and the study size.

Articles providing insufficient clinical or laboratory documentation to confidently attribute the hematological event to the oncological treatment were excluded from the final qualitative and quantitative data synthesis. The varying methodological frameworks, study designs, and statistical tools utilized across the primary literature included in this review are systematically summarized in Table 1.

## 3. Spectrum and Incidence of Hematological Toxicities

### 3.1. Immune Checkpoint Inhibitors (ICIs)

#### 3.1.1. Overall Incidence and Mortality Burden of Hem-irAEs

Hem-irAEs represent a uniquely rare subset of toxicities compared with the much more frequently encountered ICI-induced complications, such as dermatitis, colitis, or endocrinopathies. A comprehensive review of large clinical trials estimates the overall frequency of Hem-irAEs to be approximately 3.6% for all grades, with severe (grade III–IV) events occurring in only 0.7% of treated patients [35,40,61]. While high-grade irAEs are a major clinical concern, the vast majority of hematological shifts observed during anti-PD-1 monotherapy are low-grade and manageable without permanent treatment discontinuation [29]. A detailed summary of the baseline cohort sizes, patient demographics, cancer types, and primary hematological safety findings from the landmark clinical trials and registries evaluated is presented in Table 2.

Real-world pharmacovigilance data further underscores the rarity of these events. Multiple observational studies and international safety databases consistently estimate the overall frequency of Hem-irAEs to be remarkably low, ranging from 0.5% to less than 1% among patients receiving ICIs [17,33,36,62,68,69].

These events demand intense clinical vigilance because, while their overall incidence remains below 4%, the associated mortality rate is staggeringly high, estimated to range between 12% and 15.5% [35,44]. This severe mortality burden firmly establishes Hem-irAEs as one of the most perilous categories of immune-related toxicities in modern melanoma therapy. Unlike the severe and unpredictable hematological dysregulation frequently seen with dual checkpoint inhibitors, early-phase trials of liposomal melanoma vaccines have reported an absence of dose-limiting marrow suppression [6].

#### 3.1.2. The Spectrum of Hematological Manifestations

Within the rare subset of Hem-irAEs, certain conditions emerge more frequently than others. The most common clinical presentations encountered are ITP, AIHA, and neutropenia. Data derived from extensive pharmacovigilance registries and observational studies indicate that each of these three distinct entities accounts for approximately a quarter of all reported hem-irAE cases [33,34,35,70].

Beyond these relatively more frequent cytopenias, the spectrum of Hem-irAEs also encompasses a range of exceptionally rare and catastrophic clinical syndromes. During treatment with ICIs, clinicians may encounter severe, life-threatening manifestations such as AA, pancytopenia, PRCA, HLH, TTP, and acquired hemophilia A [6,17,30,35].

Furthermore, the clinical picture can be significantly complicated by the occurrence of overlapping toxicities presenting concurrently within the same patient. These complex presentations can manifest as simultaneous distinct disorders, such as ITP combined with AIHA, or as complex dual-mechanism bone marrow failures where peripheral autoantibody-mediated destruction coincides with central T-cell-mediated precursor aplasia [71]. Such overlapping events underscore the intricate and multifaceted nature of immune dysregulation induced by these therapies.

#### 3.1.3. Agent-Specific Toxicity Profiles: PD-1/PD-L1 vs. CTLA-4 Inhibitors

The risk and frequency of Hem-irAEs vary considerably depending on the specific administered ICI class. Comparative analyses of incidence rates demonstrate that the frequency of all-grade Hem-irAEs is significantly higher with therapies targeting the PD-1/PD-L1 pathway than with CTLA-4 blockade. First, there is a fundamental difference in the primary site of immune activation and phase of action between these checkpoint pathways. While CTLA-4 operates early in the immune cascade, primarily within secondary lymphoid organs (lymph nodes) to modulate T-cell priming and proliferation, the PD-1/PD-L1 axis acts later at the peripheral tissue level and within the tumor microenvironment to control T-cell effector functions and prevent chronic exhaustion [12,16,72]. Because the hematopoietic niche in the bone marrow represents a highly vascularized peripheral tissue, it is inherently more sensitive to the local, late-stage effector T-cell disinhibition triggered by anti-PD-1/PD-L1 blockade [33,35].

Second, the cellular targets of these pathways differ significantly. CTLA-4 expression is restricted almost exclusively to CD4+ and CD8+ T cells, particularly regulatory T cells (Tregs) [12,73]. Conversely, PD-1 is expressed much more broadly across the immune landscape, representing a key regulatory molecule on T cells, B cells, natural killer (NK) cells, dendritic cells, and macrophages [12,32].

Consequently, anti-PD-1/PD-L1 agents have a direct and profound impact on humoral immunity and autoantibody production. Blockade of PD-1 on B cells directly unleashes them from inhibitory control, driving clonal expansion and hyperactivation of autoreactive B-lymphocytes and plasmablasts [12,45]. This direct humoral stimulation—complemented by indirect activation of B cells via helper T cells—dramatically increases the synthesis of pathogenic autoantibodies targeting erythrocytes, platelets, or neutrophil precursors, thereby triggering peripheral autoimmune cytopenias like AIHA and ITP more frequently than anti-CTLA-4 therapies [23,34,45].

In contrast to PD-1 inhibitors, anti-PD-L1 agents may theoretically offer a distinct safety profile by allowing the physiological interaction between PD-1 and PD-L2, potentially mitigating certain severe immune-related toxicities [46]. Phase 1b clinical trials evaluating anti-PD-1 agents in distinct populations, such as Japanese patients with advanced melanoma, have reported severe, grade 3 or higher treatment-related anemia in nearly 5% of participants, underscoring the need for hematologic monitoring even with single-agent therapies [40].

Beyond overall incidence, distinct condition-specific associations exist between these drug classes and certain hematological manifestations. For example, AIHA occurs much more frequently in patients treated with PD-1 or PD-L1 targeting agents. The estimated incidence of AIHA with these specific therapies ranges from 0.15% to 0.25%, whereas the risk is substantially lower, at approximately 0.06%, for patients receiving CTLA-4 inhibitors [17,46,74]. Comprehensive systematic reviews further elucidate that while PD-1 and PD-L1 inhibitors carry heightened risks for respiratory and endocrine toxicities, they present a significantly lower risk for severe hematological suppression than traditional chemotherapy [68].

Conversely, other severe hematological syndromes present a contrasting risk profile. HLH, a catastrophic and potentially fatal condition characterized by excessive macrophage and T-cell activation, is frequently linked to anti-CTLA-4 treatment. Although secondary HLH can occur across all therapeutic classes of ICIs, clinical guidelines and pharmacovigilance data consistently indicate that it develops more commonly following anti-CTLA-4 therapy than with anti-PD-1 or anti-PD-L1 agents [12,17,39,75]. While CTLA-4 inhibitors like ipilimumab are predominantly associated with colitis and endocrinopathies, they can unpredictably trigger catastrophic autoimmune pancytopenia that requires months of careful monitoring to resolve [38]. While overall hematological toxicities for monotherapies might seem low, ipilimumab has been reported to independently induce isolated, severe thrombocytopenic events that require immediate clinical intervention [39]. Clinical experience demonstrates a distinct temporal pattern for irAEs, particularly with ipilimumab, where skin manifestations typically appear first, followed sequentially by gastrointestinal, hepatic, and eventually late-onset endocrine toxicities [76].

In evaluating fully human monoclonal PD-1 antibodies such as cemiplimab, clinical data confirm a comparable safety profile to other PD-1 inhibitors with a notably low risk of inducing persistent anti-drug antibodies [28]. In the adjuvant setting for resected stage III melanoma, patients receiving anti-PD-1 therapy exhibit a much lower rate of severe treatment-related adverse events, including leukopenia, than historical standard regimens like high-dose interferon alfa-2b [4]. In younger patient cohorts receiving checkpoint inhibitors, hematologic toxicities such as anemia are frequently reported among the most common all-grade treatment-related adverse events [69]. Despite prolonged exposure to PD-1 inhibitors like pembrolizumab, severe treatment-related cytopenias such as grade 3 to 4 anemia and neutropenia remain exceedingly rare compared with the myelosuppressive rates of traditional cytotoxic drugs [10].

Despite the severity of rare autoimmune hematologic events, the overall risk profile of PD-1 blockade makes it a statistically safer alternative to cytotoxic agents regarding widespread bone marrow suppression [62]. Systematic reviews assessing the safety of PD-L1 inhibitors such as atezolizumab reveal that the drug-related death rate is substantially lower than that of chemotherapeutics, although severe hematological events still contribute to the overall toxicity profile [62].

Additionally, toxicity manifestations often vary depending on the primary tumor, with lung cancer patients exhibiting a higher incidence of pneumonitis while melanoma patients display greater susceptibility to cutaneous toxicities and vitiligo [68]. Data from Chinese patient cohorts treated with novel anti-PD-1 agents such as tislelizumab indicate that mild-to-moderate anemia remains among the most frequently reported all-grade adverse events irrespective of the solid tumor type [29]. Furthermore, the unique distribution of melanoma subtypes in specific ethnic cohorts, such as the relatively high prevalence of acral lentiginous and mucosal melanomas in Asian populations, may influence the overall safety and efficacy profiles observed during PD-1 blockade [40].

#### 3.1.4. The Impact of Combination Immunotherapy

By blocking multiple distinct inhibitory pathways at once, the synergistic approach of dual checkpoint blockade generates a much more robust systemic immune activation; consequently, this mechanism inherently escalates the overall incidence and grade of hematological toxicities compared with monotherapy [7,13,70,77,78,79,80,81,82].

Providing statistical context to this amplified risk, clinical data demonstrate that the rate of severe (grades 3–4) toxicities overall jumps to between 55% and 59% with combined ipilimumab and nivolumab, compared with much lower rates of 16% to 28% typically seen when these agents are administered as single agents [13,25]. This steep increase in high-grade adverse events frequently necessitates early treatment discontinuation and aggressive immunosuppressive intervention. While the combination of ipilimumab and nivolumab enhances anti-tumor efficacy, it dramatically escalates the incidence of severe, grade 3–4 irAEs, often culminating in premature treatment discontinuation [75]. The comparative characteristics, all-grade and severe incidence rates, and clinical profiles across different single-agent and combination immunotherapy regimens are summarized in Table 3.

This amplified risk profile extends specifically to hematological toxicities as well. Systematic analyses reveal that the use of PD-1 inhibitors in combination with anti-CTLA-4 agents carries a significantly higher risk of all-grade cytopenias, including anemia, leukopenia, neutropenia, thrombocytopenia, and high-grade thrombocytopenia, than monotherapy [63]. Furthermore, large-scale pharmacovigilance data indicates that patients treated with anti-PD-1 in combination with anti-CTLA-4 have disproportionately higher reporting odds for severe, potentially life-threatening ITP compared with most ICI monotherapies [59]. Moreover, treatment with novel engineered IL-2 cytokines, such as nemvaleukin alfa, can induce transient, high-grade neutropenia in over half of patients with advanced melanoma [70].

Extensive real-world pharmacovigilance data confirm that dual immune checkpoint blockade generates significantly stronger positive reporting signals for severe multiorgan toxicities, including acute kidney injury and myocarditis, than single-agent therapies [78]. When ICIs are combined with targeted therapies or chemotherapy, the rates of severe toxicities, including hematological adverse events, can be notably increased compared to monotherapy [27]. Adoptive cell therapy strategies utilizing tumor-infiltrating lymphocytes, when combined with preconditioning regimens like low-dose interferon-alpha, deliberately induce transient leukopenia and neutropenia to support T-cell engraftment [82]. When novel interleukin-1 receptor accessory protein blockades are combined with pembrolizumab, clinicians must still closely monitor for hematologic dose-limiting toxicities (DLTs) such as grade 3 febrile neutropenia [37]. Advanced cellular immunotherapies, such as tumor-infiltrating lymphocytes (TILs) administered after lymphodepleting chemotherapy, carry near-universal rates of high-grade hematological toxicities, including severe febrile neutropenia [7]. While modern ICIs present distinct autoimmune toxicities, other cellular-based immunotherapeutic approaches have been prospectively evaluated across various malignancies and generally demonstrate differing, often milder, adverse event kinetics [21].

### 3.2. Targeted Therapies and Chemotherapy

#### 3.2.1. Traditional Cytotoxic Chemotherapy: Predictable Myelosuppression

Unlike the immune-mediated mechanisms of ICIs, traditional cytotoxic and cytostatic agents directly target and damage rapidly dividing hematopoietic stem cells in the bone marrow. This direct cytotoxic effect leads to predictable, dose-dependent myelosuppression and resulting cytopenias across blood lineages [9,15,50,61,87,88,89,90].

To establish a baseline, comparing the incidence of hematological toxicities between chemotherapy and immunotherapy reveals that conventional chemotherapy carries a vastly higher risk for both all-grade and high-grade hematologic toxicities. Systematic analyses show that all-grade neutropenia occurs in approximately 16.1% of chemotherapy patients versus a mere 0.5% in patients treated with PD-1/PD-L1 inhibitors. Similarly, the risk of all-grade anemia occurs in 16.2% of patients undergoing chemotherapy versus 3.4% of those receiving immunotherapy [3,4,10,51,60,62,68]. The concurrent use of antiangiogenic VEGFR inhibitors, like apatinib, alongside alkylating agents such as temozolomide presents a distinct risk for cumulative myelosuppression, occasionally culminating in grade 4 thrombocytopenia [50].

This predictable myelosuppressive profile is well-documented for specific chemotherapy regimens historically or currently used in the treatment of advanced melanoma, such as dacarbazine, paclitaxel, and carboplatin. For example, treatment episodes involving dacarbazine and paclitaxel are associated with a significantly greater percentage of hematologic adverse events (AEs) than targeted therapies like vemurafenib [2,91]. In preclinical safety assessments, emerging targeted agents generally spare the bone marrow compartment in stark contrast to traditional chemotherapies, and sometimes even induce a mild compensatory increase in hemoglobin concentrations [87]. A phase II study evaluating second-line combinations like docetaxel and carboplatin in melanoma resulted in severe (grade 3/4) neutropenia in 50% of patients, alongside grade 3/4 thrombocytopenia (13.3%) and anemia (10%) [1]. Similarly, trial data for carboplatin and paclitaxel regimens demonstrate high rates of severe hematologic toxicities, prominently featuring severe neutropenia (49%) and leukopenia (34%) [85,91].

Conversely, specialized formulations of classical chemotherapeutic agents, such as vincristine sulfate liposome injections, have demonstrated a remarkably low incidence of severe hematologic toxicity when administered for metastatic melanoma [51]. Clinical trials evaluating novel investigational agents against standard paclitaxel in second-line settings often underscore the challenging safety profiles and frequent grade 3–4 myelosuppression associated with both experimental and traditional chemotherapies [91].

Novel antibody-drug conjugates targeting melanoma-associated antigens, such as GPNMB, have also demonstrated significant bone marrow toxicity, with nearly half of treated patients experiencing grade 3 or 4 neutropenia [3].

Comprehensive meta-analyses have decisively established that PD-1 inhibitors like nivolumab carry a significantly lower relative risk for causing anemia, neutropenia, and leukopenia when compared directly to traditional chemotherapy regimens [62].

#### 3.2.2. Targeted Therapies: BRAF and MEK Inhibitors

The toxicity profile of targeted therapies contrasts significantly with the predictable myelosuppression of chemotherapy and the autoimmune-like toxicities of ICIs. While BRAF inhibitors (such as vemurafenib and dabrafenib) and MEK inhibitors (such as trametinib and cobimetinib) are most frequently associated with cutaneous toxicities including rash, keratoacanthoma, and cutaneous squamous cell carcinoma as well as cardiovascular events like hypertension, hematological shifts still represent a notable clinical challenge during treatment [2,86,87,90,92].

Real-world incidence data evaluating the safety of targeted therapies indicate that anemia and increased liver aminotransferases are among the most commonly reported adverse events in patients treated with BRAF inhibitors [93]. Notably, the development of severe (grade 3 or higher) anemia and, though more rarely, thrombocytopenia, have been documented as significant causes for unacceptable toxicity leading to treatment interruption or discontinuation, particularly in patients receiving vemurafenib [93]. Interestingly, while BRAF inhibitors are generally associated with mild cytopenias in melanoma patients, preclinical models suggest they can paradoxically enhance erythropoiesis by amplifying MAPK signaling in BRAF wild-type cells, offering a potential protective effect against concurrent anemia [90].

Beyond these more typical cytopenias, clinicians must remain vigilant for rare, severe, and potentially life-threatening hematological syndromes. For instance, secondary HLH, a fulminant syndrome characterized by sudden cytopenia, hypertriglyceridemia, and exceptionally high ferritin levels, has been reported in advanced melanoma patients receiving first-line targeted combination therapy with dabrafenib plus trametinib [94]. The emergence of this catastrophic systemic hyperinflammatory state necessitates the immediate discontinuation of the offending targeted therapy and prompt intervention with corticosteroids and supportive care to prevent a fatal outcome [94]. Novel compounds targeting NRAS-mutant melanomas, such as 4-nerolidylcatechol, have shown promising in vivo efficacy without precipitating significant peripheral leukocyte depletion [87]. Similarly, epigenetic targeted therapies evaluated in melanoma, such as HDAC inhibitors, have demonstrated challenging safety profiles characterized by frequent and severe drug-related thrombocytopenia that often necessitates treatment interruption [53,92].

The concurrent targeting of MEK and CDK4/6 pathways in NRAS-mutant melanoma yields manageable safety profiles but requires close monitoring for predictable cytopenias, including treatment-emergent anemia and neutropenia [86].

#### 3.2.3. Amplified Toxicities in Combination Regimens (Chemo-Targeted Approaches)

Analyzing the hematological impact of combining targeted agents with traditional chemotherapy reveals a significant amplification of bone marrow suppression. While single-agent BRAF or MEK inhibitors typically present with milder hematological shifts, integrating them with cytotoxic regimens heavily compounds the myelosuppressive burden on patients, frequently resulting in severe DLTs during phase I and II clinical trials [53,89,92].

For example, clinical evaluation of the BRAF inhibitor vemurafenib combined with carboplatin and paclitaxel yielded significant hematologic DLTs, including severe grade 4 thrombocytopenia [84]. In this chemo-targeted approach, grade 3 or 4 neutropenia and grade 3 or 4 thrombocytopenia each occurred in over 26% of treated patients [84]. This profoundly amplified toxicity profile frequently required aggressive clinical interventions, including dose reductions in the chemotherapeutic agents, the prophylactic or therapeutic use of granulocyte colony-stimulating factor (G-CSF), and the administration of red blood cell or platelet transfusions [84]. Despite the myelosuppressive burden of combination therapies, emerging evidence indicates that the administration of BRAF inhibitors may demonstrate a capacity to mitigate chemotherapy-induced myelosuppression, such as cisplatin-induced anemia, by promoting the self-renewal of erythroid progenitors [90].

Similarly, combining the MEK inhibitor selumetinib with standard cytotoxic drugs like docetaxel or dacarbazine produced prominent dose-limiting hematological toxicities, such as severe grade 4 thrombocytopenia and grade 4 neutropenia with fever [95]. These high-grade adverse events clearly underscore the compounding suppressive effect these combinations exert on the bone marrow reserve, demonstrating that while targeting multiple pathways may enhance tumor response, it drastically narrows the hematological therapeutic window [86,95,96].

Systematic reviews further reveal that adding anti-angiogenic agents like bevacizumab to standard chemotherapy regimens in melanoma patients regularly induces predictable myelosuppressive events, including high-grade leukopenia and thrombocytopenia [9]. Furthermore, in advanced therapeutic protocols involving adoptive cell therapy, the requisite non-myeloablative conditioning chemotherapy uniformly induces severe, albeit transient, bone marrow toxicity including prominent grade 3 to 4 neutropenia [88].

Phase I studies evaluating targeted AKT inhibitors in combination with docetaxel or carboplatin/paclitaxel have reported DLTs such as severe febrile neutropenia, necessitating careful dose modifications [89]. The combination of epigenetic modulators, such as DNA hypomethylating agents, with pembrolizumab can induce significant myelosuppression, with severe neutropenia reported in over a third of treated patients [96]. Engineered cytokine therapies, such as the intermediate-affinity IL-2 receptor activator nemvaleukin alfa, exhibit a manageable toxicity profile but can still precipitate high-grade neutropenia and cytokine release syndrome (CRS) when evaluated in solid tumors [80].

## 4. Clinical Characterization of Specific Hematological Events

An overview of the clinical prevalence, median onset kinetics, corticosteroid response rates, salvage strategies, and final outcomes for each specific hematological adverse event is presented in Table 4.

### 4.1. Red Blood Cell Disorders

#### 4.1.1. Autoimmune Hemolytic Anemia (AIHA): Clinical and Laboratory Profile

Clinical reports frequently describe checkpoint inhibitor-induced AIHA as a warm agglutinin disease, which can present as a severe and rapidly progressive hemolytic crisis [97]. AIHA represents one of the most prominent Hem-irAEs encountered in clinical practice [97]. Epidemiological data extracted from extensive pharmacovigilance databases indicate that the risk of developing AIHA is distinctly higher with therapies targeting the PD-1 or PD-L1 pathways. Specifically, the estimated incidence of AIHA with PD-1 or PD-L1 inhibitors ranges from 0.15% to 0.25%, compared with a much lower incidence of approximately 0.06% for patients receiving CTLA-4 inhibitors [74,98]. In some instances, pembrolizumab-induced AIHA can present as a life-threatening crisis complicated by concurrent immune-mediated organ injury, such as cholangiopathy [99].

The typical clinical presentation of ICI-induced AIHA is characterized by rapid clinical deterioration. Affected patients often present acutely with severe fatigue, progressive dyspnea, and physical signs of active hemolysis such as mild conjunctival jaundice [45,98].

The hallmark laboratory profile of this condition confirms a destructive hemolytic process, featuring an abrupt and severe decrease in hemoglobin levels. This profound anemia is typically accompanied by an elevated reticulocyte count, indicating an appropriate regenerative bone marrow response, alongside significantly increased lactate dehydrogenase (LDH) levels, indirect hyperbilirubinemia, and collapsed or undetectable serum haptoglobin levels [98,99,100,101].

Diagnostic confirmation heavily relies on the Direct Antiglobulin Test (DAT, or Coombs test), which is frequently found positive for IgG and/or the complement component C3d. This specific immunological profile primarily identifies the condition as a warm antibody-mediated AIHA [45,102]. Pathophysiologically, it is hypothesized that the onset of AIHA involves the unmasking or exacerbation of subclinical, pre-existing red blood cell alloantibodies or autoantibodies. This autoimmune flare is considered a direct consequence of the disruption and loss of peripheral tolerance induced by immune checkpoint blockade [57,102]. In some instances of pembrolizumab-induced AIHA, patients present with an abrupt, transfusion-dependent drop in hemoglobin alongside a positive direct Coombs test, requiring immediate cessation of immunotherapy [101].

**Table 4:** Clinical characteristics, onset kinetics, and resolution outcomes of specific Hem-irAEs.

Specific Hem-irAE	Prevalence Among Hem-irAEs (%)	Median Onset (Weeks)	Response to First-Line Steroids	Key Salvage/Second-Line Treatments	Resolution/Mortality Rate (%)	Refs.
ITP	29.0%	5.5 weeks	High (~70–80%)	IVIG, Rituximab, TPO-RAs: Romiplostim, Eltrombopag.	Resolution: ~90% Mortality: Low (~2–5%)	[1,2,3,4,5,6]
AA/pancytopenia	19.0%	10.0 weeks	Extremely Poor (<10%)	Cyclosporine A, ATG, G-CSF.	Resolution: ~20–30% Mortality: ~33–54.5%	[33,38,67,103,104,105]
Immune neutropenia	17.0%	10.5 weeks	Moderate (~50%) High when paired with G-CSF	G-CSF (Filgrastim), IVIG, Cyclosporine A, Mycophenolate Mofetil.	Resolution: ~85–90% Mortality: ~5% (sepsis risk)	[15,36,73,106,107,108]
AIHA	16.0%	7.0–10.0 weeks	Moderate (~60–70%)	IVIG, Rituximab, Cyclosporine A.	Resolution: ~75–80% Mortality: ~10%	[45,57,74,97,98,99,100,102]
HLH	11.0%	6.0 weeks	Poor as monotherapy	Etoposide (HLH-94 protocol), IVIG, Mycophenolate Mofetil, Tocilizumab, Infliximab.	Resolution: ~50–60% Mortality: High (~40–50%)	[43,55,94,109]
PRCA	8.0%	7.0 weeks	Poor (~30%)	Cyclosporine A (highly effective), IVIG, Plasmapheresis.	Resolution: ~80% (with CSA) Mortality: Low (~10%)	[42,44,110,111]

**Abbreviations:** AA, aplastic anemia; AIHA, autoimmune hemolytic anemia; ATG, antithymocyte globulin; CSA, cyclosporine A; G-CSF, granulocyte colony-stimulating factor; Hem-irAE, hematological immune-related adverse event; HLH, hemophagocytic lymphohistiocytosis; ITP, immune thrombocytopenia; IVIG, intravenous immunoglobulin; PRCA, pure red cell aplasia; TPO-RA, thrombopoietin receptor agonist.

The severity of these hemolytic events must be closely monitored. Although prompt corticosteroid therapy is the standard initial step, clinicians must be prepared for life-threatening, steroid-refractory cases of AIHA that necessitate advanced rescue immunosuppression [45,100]. Furthermore, long-term clinical management is complicated by the fact that recurrence of severe AIHA upon rechallenge with checkpoint inhibitors remains a significant and perilous risk for these patients.

#### 4.1.2. Pure Red Cell Aplasia (PRCA): Central Bone Marrow Failure

In contrast to AIHA, which is characterized by the peripheral destruction of circulating red blood cells, PRCA represents an immune-mediated central bone marrow failure that specifically targets the erythroid lineage [66]. ICI-induced PRCA is generally identified as an early-onset complication, with clinical symptoms and severe anemia typically presenting at a median of 63 to 89 days following the initiation of immunotherapy [17,42].

Establishing the diagnosis of PRCA requires careful laboratory and histological evaluation. The clinical diagnostic criteria consist of severe normocytic, normochromic anemia characterized by profound reticulocytopenia, coupled with a bone marrow biopsy (BMB) demonstrating a marked reduction or complete absence of erythroblasts in an otherwise normocellular bone marrow, preserving normal lymphoid and megakaryocytic cell lines [44,111]. Bone marrow examinations characteristically show a CD8+ T-lymphocytic infiltrate, reflecting a targeted autoimmune destruction specifically directed against erythroid precursors [42,71].

The clinical response profile of ICI-induced PRCA further underscores its specific cellular immune mechanism. Although corticosteroids are typically administered initially, the heavily T-cell-mediated nature of PRCA often renders it refractory to this approach, making targeted T-cell inhibitors a highly relevant and necessary alternative [42,110].

#### 4.1.3. Complex and Overlapping Presentations (Dual-Mechanism Anemia)

The diagnosis and management of Hem-irAEs are frequently complicated by the presence of multiple, overlapping pathobiological mechanisms occurring simultaneously within the same patient. Notably, clinicians may encounter cases of complex, dual-mechanism immune-related anemia where peripheral autoantibody-mediated red blood cell destruction (AIHA) occurs concurrently with a central T-cell-mediated failure of marrow erythropoiesis (PRCA) [66,71].

Recognizing this overlap requires careful evaluation of standard laboratory parameters. The primary clinical clue for this specific dual-mechanism toxicity is a patient who presents with a positive DAT and signs of hemolysis strongly suggesting AIHA but who exhibits paradoxical reticulocytopenia [71]. In a typical isolated case of AIHA, the bone marrow mounts a robust compensatory regenerative response, resulting in elevated reticulocytes; the absence of this response, or an inappropriately low reticulocyte count, indicates a concurrent failure in the marrow’s production of red cell precursors [71]. These clinical observations highlight a concurrent failure in the marrow’s production of red cell precursors [44,71], underscoring the critical diagnostic value of performing a bone marrow biopsy to confirm central defects, as detailed further in Section 6.1.3.

### 4.2. Platelet Disorders

#### 4.2.1. Immune-Related Thrombocytopenia (Ir-TCP) and ITP

Immune-Related Thrombocytopenia (Ir-TCP) is recognized as one of the most common Hem-irAEs, although its absolute incidence remains notably rare, estimated to be well under 1% in large melanoma patient cohorts [34,77]. However, clinicians must remain vigilant, as the risk of developing this complication significantly increases with the administration of dual checkpoint blockade (such as the combination of ipilimumab plus nivolumab) compared with ICI monotherapy [59]. Severe immune-mediated thrombocytopenia can develop rapidly after the initial infusions of PD-1 blocking agents such as nivolumab or pembrolizumab, leading to clinically significant bleeding risks [112].

The median time to onset for ir-TCP is typically around 70 days, though it can range broadly from a few weeks to several months following the initiation of treatment, and uniquely delayed occurrences have been documented even after the complete cessation of immunotherapy [34,77,112]. Clinical manifestations are highly variable; while some patients present merely with asymptomatic drops in platelet counts discovered on routine laboratory assessments, others can rapidly develop severe and potentially life-threatening symptoms, including extensive mucocutaneous bleeding, petechiae, and severe bruising [49,113].

Pathophysiologically, ir-TCP closely mimics classical ITP, as it is primarily driven by the peripheral destruction of platelets mediated by the production of autoantibodies and underlying T-cell dysregulation [59,77]. Given the lack of specific predictive biomarkers, ir-TCP remains a diagnosis of exclusion. In this context, a BMB is often an instrumental component of the diagnostic workup. A BMB is instrumental in these cases; by revealing a normal or hypercellular marrow with increased megakaryocytes, it confirms peripheral destruction over a central etiology [34,77].

#### 4.2.2. Acquired Amegakaryocytic Thrombocytopenia (AAT)

In contrast to typical ir-TCP, which is primarily driven by the peripheral destruction of circulating platelets, AAT represents a much rarer, central hematologic disorder. It is defined by severe thrombocytopenia and the complete or nearly complete absence of megakaryocytes in the bone marrow [41]. This central defect is driven by a T-cell-mediated process, with bone marrow biopsies revealing CD8+ T-lymphocytes that specifically assault and suppress megakaryocytopoiesis [41].

The clinical course of AAT is exceptionally perilous. Patients who develop AAT often exhibit severe, life-threatening drops in their platelet counts, frequently presenting with levels ≤ 5–10 × 10^9^/L and carrying a high risk of catastrophic bleeding [41]. A critical management challenge is that, unlike patients with typical ir-TCP, those with AAT generally respond poorly to standard first-line ITP therapies, showing little to no improvement with systemic corticosteroids or intravenous immunoglobulin (IVIG) [41]. Given this pronounced refractoriness to conventional treatments, alternative and targeted therapeutic strategies are required. Clinical data demonstrate that oral thrombopoietin receptor agonists (TPO-RAs), such as eltrombopag, and potent T-cell-directed immunosuppressants like cyclosporin have shown significant efficacy in restoring platelet counts and achieving complete clinical responses in these patients [41].

#### 4.2.3. Thrombotic Thrombocytopenic Purpura (TTP)

TTP is a catastrophic and highly lethal microangiopathic syndrome. It is primarily driven by acquired inhibitory autoantibodies directed against ADAMTS-13, a circulating metalloprotease responsible for cleaving large, hyperreactive von Willebrand factor (vWF) multimers into smaller, less active units [25,83]. The resulting severe deficiency in ADAMTS-13 activity allows uncleaved ultra-large vWF multimers to accumulate in the blood, where they bind to platelets and lead to the widespread formation of platelet-rich microthrombi in small vessels, causing severe end-organ microvascular injury [25,83].

While the classic clinical “pentad” of TTP consisting of thrombocytopenia, microangiopathic hemolytic anemia (MAHA), neurologic symptoms, renal failure, and fever is rarely observed in its entirety, ICI-induced TTP typically presents with an acute and deteriorating clinical picture [114]. The hallmark manifestations include profound thrombocytopenia and MAHA featuring prominent schistocytes on peripheral blood smears, accompanied by significantly elevated LDH and collapsed haptoglobin levels [25,114]. Clinically, affected patients frequently exhibit severe neurological symptoms, such as slurred speech, confusion, transient ischemic attacks, or generalized motor seizures, alongside signs of acute kidney injury [25,114,115].

In the context of immunotherapy, TTP has been prominently reported in association with anti-CTLA-4 therapy (ipilimumab) as a single agent, and with combined ipilimumab and nivolumab regimens [83,114,116]. The onset of this severe complication is typically rapid, often presenting within just weeks or a few cycles following the administration of the offending immunotherapeutic drugs [83,114].

Diagnostically, there is a critical urgency to differentiate true TTP from ITP or cancer-related MAHA. This distinction is vital because, unlike cancer-related MAHA, which generally shows normal or only modestly reduced ADAMTS-13 levels (e.g., 35–84%), TTP exhibits severely suppressed ADAMTS-13 activity (<10%) and requires immediate, life-saving therapeutic plasma exchange (TPE) [25]. Furthermore, ICI-induced TTP can present unique therapeutic challenges, as it may be exceptionally refractory to standard interventions like TPE and systemic corticosteroids [115]. In such refractory or relapsing cases, aggressive escalation of immunosuppressive therapy is often required, incorporating agents like the anti-CD20 antibody rituximab, plasma cell-targeting therapies such as bortezomib, or the targeted anti-vWF nanobody caplacizumab [83,115]. Clinicians should also note that the overall incidence of cancer-associated venous thromboembolism is notable in stage-IV patients undergoing ICI therapy, with pre-existing anticoagulation serving as a significant risk factor [52].

### 4.3. White Blood Cell Disorders

#### 4.3.1. Immune-Related Neutropenia (Ir-Neutropenia) and Agranulocytosis

Immune-Related Neutropenia (Ir-neutropenia) and agranulocytosis are exceedingly rare complications of ICI therapy, with the incidence of severe (grade 4) events estimated at only 0.14% among ICI-treated patients [36]. Despite its rarity, this toxicity carries a disproportionately high mortality risk reaching up to 14% in some cohorts due to the profound risk of overwhelming bacterial and fungal sepsis [15,36,82].

The median time to onset of ir-neutropenia typically ranges from 6.4 to 10.5 weeks after the initiation of ICI therapy [15,36]. It frequently presents acutely as febrile neutropenia, often accompanied by the classic triad of angina, tonsillitis, fever, and aphthous stomatitis or mucositis [36,58,76,96]. Affected patients may also exhibit severe localized infections, such as pharyngeal abscesses and erysipelas [36,107].

The proposed mechanisms driving this condition are complex and include both T-cell-mediated and antibody-driven processes [35,107]. Specifically, diagnostic findings have documented the presence of anti-neutrophil autoantibodies in patient serum, which is highly analogous to classic autoimmune neutropenia [35,107].

In specific demographic subgroups, such as the pediatric oncology population, the administration of ICIs has been significantly associated with disproportionately high reporting signals for both febrile neutropenia and CRS [58].

#### 4.3.2. Bone Marrow Findings and Diagnostic Confounders

Bone marrow evaluation is a crucial step in working up severe neutropenia to identify the underlying cellular deficits, such as a profound absence of the myeloid lineage or maturation arrest [15,73,106]. Immunophenotypic analyses frequently identify a pronounced CD8+ T-lymphocyte infiltration, which is responsible for the direct cytotoxic assault on early myeloid precursors [36,73].

A critical diagnostic challenge in these clinical scenarios is distinguishing true ICI-induced neutropenia from idiosyncratic drug reactions caused by concurrent medications. A major confounder in oncology is the frequent use of metamizole (dipyrone) for the management of cancer pain, as it is a well-known trigger for fatal drug-induced agranulocytosis [15,36]. While both ICIs and metamizole can cause profound bone marrow suppression and present with identical clinical symptoms, careful attention to the timeline can aid in the differential diagnosis. Metamizole-induced neutropenia typically occurs rapidly, with a median onset of 3 to 8 days after administration, whereas ICI-induced neutropenia generally follows a much more delayed onset [36].

#### 4.3.3. Eosinophilia and Large Granular Lymphocytosis (LGL)

An absolute increase in the eosinophil count is a frequently observed laboratory shift during ICI therapy. In the vast majority of cases, this ICI-induced eosinophilia is mild to moderate and remains entirely asymptomatic, representing a clinically non-significant event. Interestingly, this laboratory shift is not inherently negative; it is sometimes correlated with a favorable prognostic response to immunotherapy, particularly in melanoma patients receiving anti-CTLA-4 therapy, where it may serve as a predictive biomarker for long-term disease control. However, clinicians must maintain vigilance, as eosinophilia can occasionally manifest not as a benign shift, but as a component of severe, life-threatening systemic hypersensitivity reactions, such as Drug Reaction with Eosinophilia and Systemic Symptoms [26,35,72,117].

Beyond eosinophils, other rare white blood cell anomalies can occur, such as the emergence of Large granular lymphocytosis (LGL). While exceedingly uncommon, the clinical significance of LGL in the context of immunotherapy is notable when it presents alongside profound bone marrow suppression. Specifically, the abnormal proliferation of large granular lymphocytes has been distinctly documented to accompany rare cases of severe, immune-mediated neutropenia following ipilimumab therapy, underscoring the complex and varied nature of cellular immune dysregulation triggered by CTLA-4 blockade [35,107].

#### 4.3.4. Paraneoplastic Hyperleucocytosis (PH): A Paradoxical Reaction

Presenting a dramatically different white blood cell anomaly, paraneoplastic hyperleucocytosis (PH) contrasts sharply with the suppressive nature of ir-neutropenia. PH is defined as a sudden and massive elevation in the peripheral white blood cell count, often exceeding 100,000/µL, accompanied by a pronounced neutrophilia. Rather than being an infectious or direct autoimmune complication, this phenomenon is driven by the ectopic, paraneoplastic production of hematopoietic growth factors, specifically G-CSF or Granulocyte-Macrophage Colony-Stimulating Factor (GM-CSF), by the melanoma cells themselves [118].

While extremely rare in the context of malignant melanoma, PH has been documented to develop rapidly immediately following the initiation of combined immunotherapies, such as ipilimumab and nivolumab. The remarkably close temporal relationship between the administration of these agents and the massive spike in leukocytes suggests that the intense immune activation and the resulting profound cytokine shifts triggered by checkpoint blockade might precipitate this paraneoplastic cascade in susceptible patients [118].

The clinical significance of PH differs completely from that of typical hematological toxicities. Unlike ir-neutropenia, PH is not an autoimmune attack on the bone marrow; therefore, it does not respond to corticosteroids or other immunosuppressive therapies. Instead, because the paraneoplastic production of growth factors like G-CSF can lead to permanent autocrine stimulation of the melanoma cells, the emergence of PH is a harbinger of uncontrollable, rapid tumor progression. It is associated with a highly aggressive disease course and indicates a very poor prognosis with severely limited patient survival [118].

### 4.4. Bone Marrow Failure and Systemic Syndromes

#### 4.4.1. Immune-Related Aplastic Anemia and Pancytopenia

Immune-related AA and pancytopenia are recognized as among the rarest but most catastrophic and life-threatening hematological irAEs. The associated mortality is exceptionally high, with various cohorts reporting mortality rates ranging from 33% to 54.5% [35,44]. These fatalities are most frequently driven by severe and rapidly progressing complications, such as overwhelming bacterial sepsis, systemic fungal infections, or intrinsically refractory bone marrow failure [35].

Clinically, affected patients typically present with an acute triad of symptoms: severe fatigue resulting from non-regenerative anemia, prominent bleeding diatheses due to profound thrombocytopenia, and high fever or active infections secondary to agranulocytosis. This severe marrow suppression is notoriously prolonged, often persisting for months, and consistently necessitates significant and frequent red blood cell and platelet transfusion support to temporarily stabilize the patient [35,104].

Histological evaluation of the bone marrow is required to establish a definitive diagnosis of AA, typically revealing a markedly hypocellular or ‘empty’ marrow that confirms central hematopoietic failure. The pathological examination typically reveals a markedly hypocellular or “empty” marrow often demonstrating less than 10% overall cellularity accompanied by severe trilineage hypoplasia. Furthermore, immunophenotyping of the residual marrow frequently demonstrates a pronounced predominance of activated CD8+ T-lymphocytes alongside an inverted CD4+:CD8+ ratio, indicating that the fundamental pathobiology is a direct cytotoxic T-cell-mediated destruction of early hematopoietic precursors [35,67,105].

Unlike many other irAEs that respond predictably to immunosuppression, immune-related AA possesses a notoriously low clinical resolution rate, reported to be as low as 25%, and is frequently refractory to standard first-line high-dose corticosteroids [35]. Given this profound treatment resistance, there is an absolute necessity for advanced, targeted immunosuppressive strategies. Effective management often requires the incorporation of agents such as anti-thymocyte globulin (ATG), cyclosporine, and TPO-RAs, which must be strictly paired with vigilant anti-infective prophylaxis to protect these highly vulnerable patients from fatal infectious complications [17,35,67].

#### 4.4.2. Hemophagocytic Lymphohistiocytosis (HLH)/Macrophage Activation Syndrome

HLH is a severe, fulminant systemic inflammatory syndrome characterized by a devastating “cytokine storm.” This hyperinflammatory state is fundamentally driven by the excessive activation and proliferation of macrophages and cytotoxic T-cells, leading to widespread tissue injury and multi-organ dysfunction [55,94].

While HLH can emerge as a paraneoplastic condition or secondary to viral reactivations [such as the Epstein–Barr virus (EBV)], it is increasingly recognized as a rare but catastrophic complication of ICIs, encompassing both anti-PD-1 and anti-CTLA-4 therapies [30,94]. Furthermore, secondary HLH has been uniquely documented in melanoma patients treated with targeted BRAF/MEK inhibitors (e.g., dabrafenib and trametinib). In these specific cases, HLH has been observed developing rapidly following the initiation of targeted therapy or manifesting as a sequential complication after a prior failure of immunotherapy [43,94].

The clinical presentation of HLH is highly heterogeneous and non-specific, frequently overlapping with profound sepsis or rapid disease progression, which can significantly delay diagnosis [30]. To navigate this diagnostic challenge, clinicians utilize the HLH-2004 criteria and the HScore calculator to estimate diagnostic probability based on key clinical and laboratory findings. The hallmark features include unremitting high fever, hepatosplenomegaly, progressive cytopenias across ≥2 lineages, extreme hyperferritinemia (often exceeding 5000–10,000 µg/L), hypertriglyceridemia, hypofibrinogenemia, elevated soluble CD25 (IL-2 receptor), and the morphological demonstration of hemophagocytosis (activated macrophages engulfing erythrocytes, leukocytes, or platelets) in the bone marrow aspirate [35,43,55]. Crucially, histopathologic evidence of hemophagocytosis might be delayed or entirely absent initially; therefore, life-saving treatment should not be withheld pending definitive biopsy results if the clinical suspicion remains high [35,55].

HLH constitutes an absolute medical emergency, carrying a staggering mortality rate reported between 14.8% and 43% in various immunotherapy cohorts [35,43]. Survival depends on aggressive interventions. The required protocol involves the immediate discontinuation of the offending drug and the prompt initiation of high-dose intravenous corticosteroids (e.g., 2–5 mg/kg/day). Moreover, the critical early introduction of cytokine-directed therapy, particularly the Interleukin-6 (IL-6) receptor antagonist tocilizumab, has proven highly effective in bridging the inflammatory cascade [35,55]. For patients whose condition is refractory to these initial measures or who are rapidly deteriorating, the cytotoxic agent etoposide should be administered to selectively deplete the aberrantly activated T-cells and suppress the ongoing production of inflammatory cytokines [35,55,109].

## 5. Pathophysiology and Risk Factors

### 5.1. Immune Mechanisms

#### 5.1.1. T-Cell Hyperactivation and Central/Peripheral Tolerance Breakdown

ICIs exert their potent anti-tumor efficacy by removing inhibitory signals, primarily CTLA-4 and PD-1, which physiologically function to maintain peripheral tolerance and prevent autoimmunity [12,26]. By blocking these critical regulatory pathways, ICIs induce an unregulated activation of the adaptive immune system, leading to a profound loss of immunogenic self-tolerance and the subsequent expansion of autoreactive T-cell clones that can mistakenly target healthy tissues [12]. By specifically blocking CTLA-4, agents like ipilimumab allow CD28 to act without restriction, amplifying the immune response but concurrently diminishing self-tolerance [75].

A central component of this loss of self-tolerance involves Treg dysfunction. ICIs may actively diminish the survival and suppressive function of Treg cells, severely disrupting the functional homeostasis that normally exists between Tregs and effector T cells, such as pro-inflammatory type 17 T helper cells [12,119]. This impaired regulatory function removes the natural physiological safeguards against self-reactivity, thereby allowing unchecked autoimmune responses to proliferate and manifest as severe systemic or organ-specific irAEs [45,119]. The significance of a pre-existing positive DAT before initiating immunotherapy is a critical area of investigation, as it may serve as an early predictor for the subsequent development of fulminant AIHA [99].

In the context of central hematological toxicities, this breakdown of tolerance culminates in direct CD8+ T-cell cytotoxicity within the bone marrow. Rare and severe conditions such as immune-related AA, PRCA, and AAT are primarily driven by the direct, T-cell-mediated destruction of hematopoietic precursors [41,44,67]. This mechanism is strongly evidenced by bone marrow biopsies from affected patients, which characteristically show a marked infiltration of polyclonal CD8+ T-lymphocytes directly assaulting the marrow space, frequently accompanied by an inverted CD4+:CD8+ ratio indicating a predominantly cytotoxic autoimmune response [41,67,71].

#### 5.1.2. B-Cell Dysregulation and Autoantibody Production

While ICIs are primarily designed to target and reinvigorate T cells, the PD-1 receptor is also notably expressed on B cells, playing a significant role in their development and regulation. Consequently, the blockade of PD-1 or CTLA-4 pathways can precipitate an abnormal humoral immune response. This occurs either by directly unleashing B cells from inhibitory control or by indirectly stimulating them via the hyperactivation of CD4+ T-helper cells [45,102]. Clinical and genomic profiling studies have shown that during ICI therapy, patients often experience a decline in the overall number of circulating B cells; however, this is accompanied by a paradoxical and simultaneous expansion in the population of CD21low B cells and plasmablasts. These specific expanded subsets express autoreactive B-cell receptors and gene signatures that are strongly linked to the initiation and perpetuation of autoimmunity [12,26,119].

This profound B-cell dysregulation and subsequent autoantibody production serve as the primary pathobiological mechanism behind peripheral hematological irAEs. The generated autoantibodies inappropriately target self-antigens present on the surface of circulating blood cells, directly leading to immune-mediated peripheral destruction conditions such as AIHA, ITP, and autoimmune neutropenia [34,36,45]. Importantly, the administration of ICIs can act as a systemic catalyst that unmasks or exacerbates a wide range of pre-existing, subclinical autoantibodies by triggering the rapid proliferation of latent autoreactive B-cell clones [28,57,102].

#### 5.1.3. Cytokine Storms and Macrophage Activation

The administration of ICIs can trigger massive T-cell and B-cell activation, which in turn leads to the abnormal and excessive release of pro-inflammatory mediators. Among these critical mediators are cytokines such as interleukin-1β (IL-1β), IL-2, IL-6, Interleukin-17 (IL-17), and interferon-gamma (IFN-γ) [12,119]. This massive cascade of cytokines drives both systemic and organ-specific inflammation, actively contributing to immune-mediated tissue damage [12,26,80]. Furthermore, this heightened hyperinflammatory state and the resulting cytokine shifts can severely disrupt normal hematopoiesis, laying the pathophysiological groundwork for complex hematological adverse events [12,119]. The dual release of immune brakes inherent to combination ICI regimens can trigger a self-perpetuating cycle of systemic immune cell infiltration and massive cytokine release, directly precipitating clinical CRS [78].

This profound cytokine-mediated mechanism can ultimately culminate in catastrophic and life-threatening systemic conditions, most notably secondary HLH. ICI-induced HLH represents a severe hyperinflammatory state characterized fundamentally by the excessive activation and proliferation of macrophages and cytotoxic T cells [43,55]. This cellular overactivation triggers a devastating “cytokine storm,” frequently marked by highly elevated levels of circulating pro-inflammatory cytokines, specifically IL-6, IL-18, and CXCL9 [12,55]. Ultimately, this extreme systemic hyperinflammation drives the aberrant engulfment of erythrocytes, leukocytes, and platelets by activated macrophages, manifesting as overt hemophagocytosis within the bone marrow space [43,55].

The clinical success of anti-TNF-alpha therapies in mitigating severe steroid-refractory irAEs underscores the central role that specific pro-inflammatory cytokines play in the propagation of widespread immune toxicity [120]. Systemic administration of novel interleukin-based therapies can trigger transient CRS and associated myelosuppressive events, emphasizing the need for carefully optimized dosing schedules [80]. Following the infusion of genetically engineered T-cells, the rapid systemic elevation of pro-inflammatory cytokines such as IL-6 and IFN-γ is directly responsible for precipitating CRS and further disrupting baseline hematopoiesis [54].

#### 5.1.4. Shared Antigens, Molecular Mimicry, and Direct Off-Target Effects

Another critical mechanism driving hematological toxicities involves antigenic cross-reactivity and molecular mimicry between malignant cells and normal host tissues. The shared antigen theory postulates that tumor cells and normal hematopoietic tissues may express identical or structurally similar specific antigens [108]. When ICIs successfully stimulate the adaptive immune system to attack tumor neoantigens, the resulting massive expansion of autoreactive T cell clones can lead to an unintended “bystander” effect. These invigorated T cells may mistakenly recognize and target cross-reactive self-antigens present on healthy blood cells or bone marrow precursors, driving organ-specific autoimmunity and resulting in immune-mediated tissue damage [12,26,108].

Beyond T-cell-mediated cross-reactivity, Hem-irAEs can also be precipitated by the direct molecular off-target binding of the checkpoint inhibitors themselves. ICIs may directly bind to non-tumor cells that physiologically express the targeted immune checkpoint molecules, subsequently mediating complement-driven tissue damage or direct cellular inhibition [12]. For instance, PD-L1 is known to be expressed on human platelets; consequently, administered anti-PD-L1 monoclonal antibodies might directly bind to and inhibit these circulating platelets, acting as a mechanical and immunological contributing factor to the development of ICI-induced ITP [59]. Additionally, the administration of these agents and the subsequent immune dysregulation can trigger profound intracellular abnormalities. Recent transcriptomic analyses have linked the pathogenesis of ICI-induced ITP to the overactivation of the mammalian target of rapamycin signaling pathway, which may lead to aberrant megakaryocyte autophagy and consequent functional failure within the bone marrow [59].

### 5.2. Patient-Specific Risk Factors

#### 5.2.1. Pre-Existing Autoimmune Diseases (ADs) and Baseline Autoantibodies

Patients with pre-existing ADs, such as psoriasis, rheumatoid arthritis, inflammatory bowel disease, or Hashimoto’s thyroiditis, have traditionally been excluded from major ICI clinical trials due to significant concerns regarding excessive toxicity and autoimmune exacerbation [12,17]. However, emerging real-world data reveals that while ICIs can often be administered safely in this population, these patients face a substantially higher risk of experiencing exacerbations or flares of their underlying disease and developing *de novo* irAEs than patients without a history of autoimmunity [119,121].

Specifically regarding hematological toxicities, pre-existing hematological abnormalities serve as a major risk factor. Patients presenting with a prior medical history of anemia, AIHA, ITP, or known red blood cell alloantibodies and autoantibodies are at a markedly increased risk for developing severe, potentially life-threatening hematological irAEs upon ICI exposure [34,57]. For example, the known presence of baseline red cell alloantibodies or autoantibodies serves as a major risk factor, as the initiation of PD-1 blockade can rapidly exacerbate this underlying predisposition into fulminant clinical AIHA [57,102]. Patients with a history of other autoimmune disorders, such as Hashimoto’s thyroiditis, may be at a heightened risk for developing severe ITP during PD-1 blockade [112].

Furthermore, the presence of circulating baseline autoantibodies prior to the initiation of ICI therapy is increasingly recognized as a predictive factor for toxicity. Routine pre-treatment immunological screening that detects autoantibodies such as antinuclear antibodies (ANA), anti-thyroid antibodies, rheumatoid factor, or a positive DAT can predict the subsequent development of target-specific irAEs or the severe exacerbation of subclinical hematological conditions [12,34,57]. The presence of these baseline markers reflects an underlying systemic inflammatory state and a preexisting propensity for autoimmunity that checkpoint blockade can easily tip into overt, clinical toxicity [26,119].

#### 5.2.2. Concomitant Hematological Malignancies (The CLL Link)

A unique and significant risk factor for the development of Hem-irAEs is a past or concurrent medical history of hematolymphoid tumors, most notably B-cell chronic lymphocytic leukemia (CLL) [57]. Retrospective analyses and pharmacovigilance data have highlighted a disproportionate prevalence of these hematological malignancies among affected patients. Studies reveal that up to 9% of patients who develop severe Hem-irAEs while being treated with ICIs for solid tumors have a concomitant diagnosis of CLL, suggesting that these patients are at a markedly increased risk of hematological immunotoxicity and may benefit from closer surveillance [35,36,57,103].

The primary mechanistic hypothesis behind this phenomenon proposes that the presence of an underlying mature lymphoid B clone significantly increases the susceptibility to ICI-induced profound cytopenias, such as AIHA and severe (grade 4) neutropenia [35,36]. In these patients, checkpoint blockade may easily disrupt immune tolerance and trigger the latent autoreactive potential of these preexisting abnormal B-cell populations, leading to aggressive autoantibody production [57]. Given this pronounced risk, baseline screening for underlying CLL or monoclonal B-cell lymphocytosis may be warranted for patients prior to initiating immunotherapy. Moreover, if catastrophic events like severe hemolysis or bone marrow failure occur in this vulnerable population, standard corticosteroid therapy is often insufficient; therefore, proactive, targeted anti-B-cell therapies, such as rituximab or ibrutinib, may be required to effectively halt the immune destruction and restore normal hematopoiesis [36,57].

#### 5.2.3. Demographic and Anthropometric Factors (Age, Sex, and BMI)

Age plays a complex role in the risk profile of ICI therapies. While aging is intrinsically linked to immune senescence and alterations in the immune system that might theoretically blunt immune responses, real-world data indicates that age uniquely affects toxicity patterns [12]. Retrospective analyses suggest that older patients (≥65 years) exhibit a significantly higher incidence of specific side effects, particularly dermatologic toxicities [21,29,122]. Conversely, younger age has been associated with the development of more severe, unique, or multi-organ irAEs, often requiring acute interventions and hospitalizations [12,69]. While age-related immune senescence influences toxicity in adults, pediatric patients exhibit a unique vulnerability to specific severe manifestations, such as acute respiratory distress syndrome and immune-mediated seizures, potentially driven by distinct mechanisms of inflammatory imbalance [58]. Systematic reviews of pediatric, adolescent, and young adult populations demonstrate that immune checkpoint blockade maintains a comparable safety profile to that seen in adults, though careful monitoring for severe adverse events remains necessary [69].

The influence of patient sex on irAE susceptibility presents conflicting data depending on the specific toxicity being analyzed. Broader pharmacovigilance and real-world studies frequently indicate that female patients face a higher overall risk for developing severe irAEs, a trend potentially driven by hormonal influences and higher baseline predispositions to primary autoimmunity [12,58,64]. However, specific data focusing on hematological irAEs reveal an inverse pattern. Conditions such as ITP and AIHA demonstrate a distinct male predominance, with cohorts consistently reporting male patient proportions ranging from 58% to over 61% in these hematological events [56,59].

Anthropometric factors also significantly predict toxicity risks. A higher body mass index (BMI) and clinical obesity are independently associated with significantly increased odds of developing irAEs across various cancer types [119,122]. Conversely, but equally important, poor baseline performance status factors such as low muscle mass (sarcopenia) have been distinctly linked to the onset of high-grade irAEs, particularly observed in melanoma patients undergoing treatment with anti-CTLA-4 therapy like ipilimumab [122,123].

#### 5.2.4. Genetic Susceptibility (HLA Types and Polymorphisms)

The host genetic background, particularly concerning Human Leukocyte Antigens (HLA), is increasingly recognized for its role in predisposing patients to distinct autoimmune toxicities following immunotherapy. Specific HLA genotypes appear to act similarly to primary autoimmune disease risk loci, tipping the balance toward loss of self-tolerance when immune checkpoints are blocked. For example, autoimmune disease-associated susceptibility loci such as the HLA-DR4 allele demonstrate a striking predominance in patients who develop ICI-induced insulin-dependent diabetes, while HLA-DRB1 alleles (such as HLA-DRB1*04:05) are strongly associated with the onset of ICI-induced inflammatory arthritis [12,26]. Additionally, genetic variants in fundamental DNA repair pathways, such as specific single nucleotide polymorphisms (SNPs) within the Fanconi Anemia pathway genes BRCA2 and FANCA, have been identified as independent predictors of OS and prognosis in melanoma patients [65].

Beyond HLA profiling, genetic variability in the targeted checkpoint genes themselves via SNPs offers another layer of predictive insight, particularly for hematological toxicities. Specific SNPs mapped to the *PDCD1* (encoding PD-1) and *CTLA4* genes have been linked to the susceptibility and clinical features of immune-related cytopenias. For instance, the PD-1 +7209 C/T SNP has been associated with an increased susceptibility to ITP, while the CTLA4 −1577 A/G polymorphism is notably linked to ITP severity [34]. These findings suggest that intrinsic genetic variants within the checkpoint pathways could serve as powerful predictive biomarkers to identify patients at a high risk for developing severe hematological toxicities prior to the initiation of therapy [34,65]. Identifying specific risk factors and utilizing predictive markers like the neutrophil-to-lymphocyte ratio (NLR) might help clinicians anticipate the heightened thrombotic burden in cancer patients undergoing immunotherapy [47].

#### 5.2.5. Baseline Laboratory and Systemic Biomarkers

Routine baseline shifts in standard blood components, particularly Complete Blood Count (CBC) indices, have emerged as accessible predictive biomarkers for immunotherapy-induced toxicity. Elevated ratios reflecting systemic inflammation, such as a high NLR and a high platelet-to-lymphocyte ratio, are associated with a significantly increased risk of developing severe irAEs [119,122,124]. Similarly, an elevated absolute eosinophil count at baseline or early in the treatment course has been significantly correlated with the onset of irAEs [26,122]. Additionally, pre-existing quantitative deficits in the blood compartment, such as low hemoglobin or low platelet counts prior to therapy initiation, independently predict poor clinical outcomes and a higher overall incidence of irAEs [31,52,122]. Conversely, higher baseline hemoglobin values within normal limits have been paradoxically linked to an increased risk of subsequent immune-related cardiotoxicity in melanoma patients receiving checkpoint blockade [31]. Baseline thrombocytosis in melanoma patients is significantly correlated with a higher likelihood of presenting with distant metastases and serves as an independent predictor of decreased OS [124]. Furthermore, unexpected and severe hematological toxicities during novel therapy administration can frequently be attributed to underlying pharmacokinetic variations and low drug clearance in specific patient subsets [91].

Beyond cellular indices, circulating pro-inflammatory cytokines and acute-phase reactants provide further insight into a patient’s predisposition to immunotoxicity. Elevated baseline levels of C-reactive protein are consistently linked to a higher risk of irAE incidence and a worse overall prognosis [122]. Furthermore, specific cytokine profiles, notably elevated baseline levels of IL-17 or altered IL-6 dynamics, reflect a preexisting systemic inflammatory state that strongly correlates with the likelihood of developing severe irAEs [17,26,37,119]. For instance, high baseline IL-17 has been specifically linked to the subsequent development of severe immune-mediated colitis, while IL-6 increases are recognized as prognostic factors for broader autoimmune toxicities [17,119]. Recent evidence suggests that chronic IL-1β-induced inflammation acts as a central catalyst for immunosuppression, driving the rationale for combining anti-IL-1RAP agents with standard PD-1 inhibitors [37]. Interestingly, high baseline expression of T-cell co-stimulatory markers, such as CD28 and CD27, has been correlated with a significantly shorter duration of treatment-induced myelosuppression, suggesting potential utility as predictive biomarkers for hematopoietic recovery [88].

#### 5.2.6. Prior Therapies and Concomitant Medications

Patients who have been heavily pre-treated with multiple lines of traditional cytotoxic chemotherapy or ionizing radiation often exhibit a significantly reduced baseline bone marrow reserve [61]. When ICIs are subsequently introduced, these patients are notably more vulnerable to developing severe, prolonged immune-mediated cytopenias due to their pre-existing hematopoietic compromise and diminished capacity for bone marrow regeneration [61].

Furthermore, evaluating concomitant medications is critical, particularly the use of metamizole (dipyrone). As a known trigger for idiosyncratic agranulocytosis, its concurrent administration significantly compounds the risk of severe marrow toxicity and complicates the clinical picture during immunotherapy [15,36]. Because the clinical presentation, timing of onset, and even bone marrow findings can overlap significantly between the two etiologies, distinguishing between a true ICI-induced immune-related adverse event and metamizole-induced marrow toxicity remains a major clinical challenge.

## 6. Diagnostic Work-Up and Management Strategies

### 6.1. Diagnostic Algorithms

#### 6.1.1. Baseline Assessment and Routine Laboratory Monitoring

Because oncologists frequently rely on standard transfusion thresholds when evaluating hematological parameters (e.g., minimizing a drop in hemoglobin until it falls below 10 g/dL), mild or early cytopenias can be easily overlooked in patients receiving immunotherapy. Therefore, it is critical to compare a patient’s on-treatment laboratory values directly to their individual baseline values established before ICI initiation. This personalized approach is essential to detect early, gradual modifications in blood counts that may signal the onset of a hematological immune-related adverse event (irAE) rather than a simple fluctuation [34,38,48,124,125]. Rigorous prospective monitoring of hematologic parameters is essential across all forms of immune-cell therapy, as it allows clinicians to safely distinguish between expected transient immunological reactions and the onset of severe systemic toxicity [21].

To capture asymptomatic drops in hemoglobin, platelets, or neutrophils, routine CBC with differential must be performed intermittently throughout the entire course of treatment [17]. Ideally, these laboratory assessments should be conducted prior to each treatment cycle. Moreover, monitoring should be escalated to weekly intervals for patients receiving high-risk combination therapies, such as ipilimumab plus nivolumab, to ensure the early detection of potentially life-threatening hematological shifts [34,126].

Any new, unexplained blood count abnormality detected during routine monitoring should trigger an immediate and comprehensive clinical evaluation. The initial workup must include an assessment of the patient’s nutritional status (including testing for iron, folate, and vitamin B12 deficiencies) and a thorough physical examination to identify underlying signs such as splenomegaly, hepatomegaly, purpura, or spontaneous bleeding [34]. Before initiating checkpoint blockade, a comprehensive evaluation of the patient’s baseline laboratory values, including CBCs and thyroid function tests, is essential to establish a reference point and quickly identify emerging toxicities [32]. Furthermore, clinicians must meticulously review all concurrent medications to definitively rule out non-immune, drug-induced cytopenias. For instance, the use of metamizole must be strictly documented during the medication review, as it can trigger acute agranulocytosis that perfectly mimics an ICI-induced event, acting as a major diagnostic confounder. [34,36]. This supports the dual utility of baseline erythrocyte and hemoglobin parameters, as they may act as predictive markers not only for bone marrow failure but also for systemic cardiovascular immune-related events [31]. While predictive models like the Khorana score are effective indicators of overall mortality in stage-IV cancer patients, their utility in specifically anticipating ICI-induced cancer-associated thrombosis appears limited [52]. Frequent monitoring is critical when utilizing engineered immunocytokines, as they can cause rapid, treatment-emergent leukopenia and infusion-related reactions [79]. The routine evaluation of platelet counts not only helps identify immune-mediated thrombocytopenia but also provides critical prognostic information, as elevated levels may reflect a paraneoplastic inflammatory state [124]. Continuous and weekly monitoring of hematologic parameters is especially mandated during phase I combination trials, as agents with both immunomodulatory and antiangiogenic properties can synergistically and unpredictably suppress the bone marrow [81]. Persistent, therapy-induced lymphopenia and neutropenia in the context of cellular immunotherapy can paradoxically serve as a marker of successful preconditioning and positively correlate with ultimate clinical response [82].

#### 6.1.2. The Tiered Diagnostic Work-Up for Peripheral Cytopenias

The peripheral blood smear serves as a rapid and indispensable first-line diagnostic tool in the evaluation of peripheral cytopenias. It is critical for identifying specific morphological abnormalities, such as red blood cell agglutination or spherocytes, which are highly suggestive of an underlying AIHA [34,100]. Additionally, the smear can reveal schistocytes, a hallmark finding of thrombotic microangiopathies such as TTP, or detect platelet clumping to quickly rule out pseudo-thrombocytopenia [34].

In the setting of acute anemia, a robust hemolysis laboratory panel is absolutely essential to determine the etiology. This work-up must include the DAT to specifically check for the presence of IgG or C3d autoantibodies bound to the red blood cell surface [17,35,57]. Alongside the DAT, the comprehensive evaluation of hemolysis requires measuring LDH, reticulocyte count, unconjugated bilirubin, and haptoglobin levels to confirm red blood cell destruction and evaluate the compensatory bone marrow response [17,35,57].

For patients presenting with suspected ITP, it is vital to perform comprehensive coagulation studies including prothrombin time, activated partial thromboplastin time (aPTT), fibrinogen, and d-dimer to definitively rule out consumptive coagulopathies like disseminated intravascular coagulation (DIC) [17]. In cases presenting with MAHA alongside thrombocytopenia, measuring the ADAMTS-13 activity level is vital to confirm a diagnosis of TTP [17,25]. Furthermore, if the catastrophic hyperinflammatory syndrome HLH is clinically suspected, the laboratory evaluation must be rapidly expanded to assess syndrome-specific markers including ferritin, triglycerides, fibrinogen, and the soluble IL-2 receptor (sCD25) [55].

Finally, the diagnostic algorithm must incorporate comprehensive infectious and autoimmune serology. It is an absolute necessity to rule out primary viral infections or subclinical reactivations such as Parvovirus B19, EBV, cytomegalovirus, human immunodeficiency virus, and Hepatitis B and C through polymerase chain reaction and serological testing. These viral pathogens can closely mimic the clinical presentation of Hem-irAEs or act as the primary trigger for the immune dysregulation [34,35].

#### 6.1.3. The Pivotal Role of Bone Marrow Biopsy (BMB)

The comprehensive evaluation of severe immune-mediated cytopenias mandates a bone marrow aspirate and core biopsy to definitively distinguish between peripheral destruction mechanisms and central marrow failure syndromes [35,103]. In peripheral destruction conditions, such as typical AIHA or ITP, the bone marrow frequently appears normal or distinctly hypercellular, reflecting a compensatory hyperplasia in response to the peripheral consumption of circulating cells [44]. In stark contrast, central marrow failure syndromes induced by immunotherapy such as PRCA, AA, and AAT characteristically present with severe lineage-specific hypoplasia or a completely empty marrow space lacking the respective hematopoietic precursors [41,44,71,88].

Furthermore, histopathological assessment using BMB is essential to exclude melanoma infiltration and can unexpectedly reveal underlying monoclonal B-cell lymphocytosis or CLL in patients presenting with ICI-induced pancytopenia [35,44,103]. Massive marrow infiltration by metastatic melanoma can present with clinical and laboratory features identical to severe irAE, including profound cytopenias or the sudden onset of DIC [47,127]. Differentiating a true immune-mediated cytopenia from malignant tumor invasion is paramount, as the two entities require entirely different management strategies, shifting the clinical approach from urgent high-dose immunosuppression to the potential initiation of alternative systemic anti-neoplastic therapies [35,127].

Finally, detailed immunohistochemical analyses of the BMB are essential to visualize the central immune pathology, frequently revealing a prominent CD8+ T-lymphocytic infiltrate with a characteristically inverted CD4+:CD8+ ratio [67,71]. Identifying these infiltrates is particularly crucial in diagnosing highly complex clinical scenarios involving dual-mechanism anemias. For instance, a patient might present with a strongly positive DAT and elevated hemolysis markers, strongly suggesting a peripheral AIHA, but simultaneously display a paradoxical reticulocytopenia [71]. In such confusing cases, a BMB is essential to reveal that the failure to adequately produce reticulocytes is due to a concurrent CD8+ T-cell-mediated central PRCA, thus necessitating a specific targeted immunosuppressive approach beyond standard corticosteroid therapy [71].

#### 6.1.4. Radiographic and Radionuclide Imaging Findings in Hem-irAEs

While hematological toxicities are primarily diagnosed via peripheral blood counts and bone marrow biopsy, medical imaging plays a crucial role in ruling out differential diagnoses, staging concomitant non-hematological toxicities, and characterizing specific systemic syndromes [16]. In autoimmune cytopenias (such as AIHA or ITP), fluorodeoxyglucose positron emission tomography (18F-FDG PET/CT) imaging typically reveals diffuse reactional osteomedullary hypermetabolism, reflecting compensatory bone marrow hyperplasia in response to peripheral destruction [110]. In contrast, central bone marrow failure syndromes (such as AA) usually lack this reactional hypermetabolism, or display focal, heterogeneous uptake due to patchy inflammatory infiltration [67]. Furthermore, in HLH, 18F-FDG-PET/CT is valuable for detecting marked hepatosplenomegaly and diffuse lymphadenopathy without focal malignant lesions, helping differentiate HLH from disease progression [55].

Abdominal ultrasound and CT are essential to evaluate for hepatosplenomegaly and rule out portal hypertension or splenic sequestration as the primary drivers of cytopenia [35]. In patients presenting with severe, acute dyspnea, CT pulmonary angiography (CTPA) is mandatory to exclude pulmonary embolism (associated with ICI-induced thrombosis) [47]. High-resolution chest CT also assists in ruling out overlapping immune-related pneumonitis (characterized by ground-glass opacities) [17,128].

Brain magnetic resonance imaging (MRI) with thin cuts of the sella turcica is indicated in cases of secondary cytopenias arising from central endocrinopathies (such as hypophysitis), which can reveal pituitary enlargement, stalk thickening, or late-stage empty sella syndrome [117,128].

### 6.2. First-Line and Refractory Management

#### 6.2.1. Initial Intervention: ICI Discontinuation, Corticosteroids, and Supportive Care

The cornerstone of initial treatment for Hem-irAEs involves the immediate interruption of the offending ICI. [18,20,75,128]. Current clinical guidelines generally recommend temporarily withholding ICI therapy for moderate, grade 2 toxicities until symptoms and laboratory values revert to grade 1 or baseline [14,17]. However, for severe (grade 3 or 4) or recurrent hematological irAEs, permanent discontinuation of the ICI is strongly advised to prevent further life-threatening immune-mediated damage [14,18]. Fortunately, while severe neutropenic events occur with engineered interleukins, they are typically manageable with dose delays and rarely lead to permanent treatment discontinuation or grade 5 adverse events [70]. Because novel targeted agents like panobinostat can induce early and significant peripheral blood cytopenias, frequent dose modifications and careful hematologic monitoring are strictly required to manage DLTs [92].

Once the ICI is interrupted, high-dose systemic corticosteroids serve as the foundational first-line pharmacological treatment for the vast majority of hematological irAEs [19,35]. Typical treatment regimens involve the administration of oral prednisone at 1 to 2 mg/kg/day for moderate manifestations [14,17]. For more severe, grade 3 or 4 toxicities where patients frequently require hospitalization, guidelines recommend the immediate initiation of intravenous methylprednisolone at 1 to 2 mg/kg/day to rapidly suppress the hyperactive immune response [14,18,32,76]. Because therapeutic monoclonal antibodies initiate complex and cascading immune responses, the systematic implementation of systemic corticosteroids forms the foundational approach to mitigate escalating collateral inflammatory damage [20,120]. For targeted agents and antibody-drug conjugates, severe treatment-emergent cytopenias are often reversible and can be effectively managed with the timely administration of GM-CSF [3].

A critical component of this immunosuppressive strategy is the subsequent steroid taper. It is of paramount importance to implement a slow and gradual steroid taper over a minimum of 4 to 6 weeks once symptoms and blood counts have improved to grade 1 or baseline [14,123]. Clinicians must closely monitor patients during this phase, as rapid withdrawal or tapering of corticosteroids frequently leads to a rebound or relapse of the hematological irAE [14,123]. In cases where immune-mediated toxicities, such as severe colitis or hepatitis, prove refractory to initial high-dose corticosteroids within a few days, the prompt addition of secondary immunosuppressants like infliximab or mycophenolate mofetil is strongly recommended [117].

**Figure 2:**
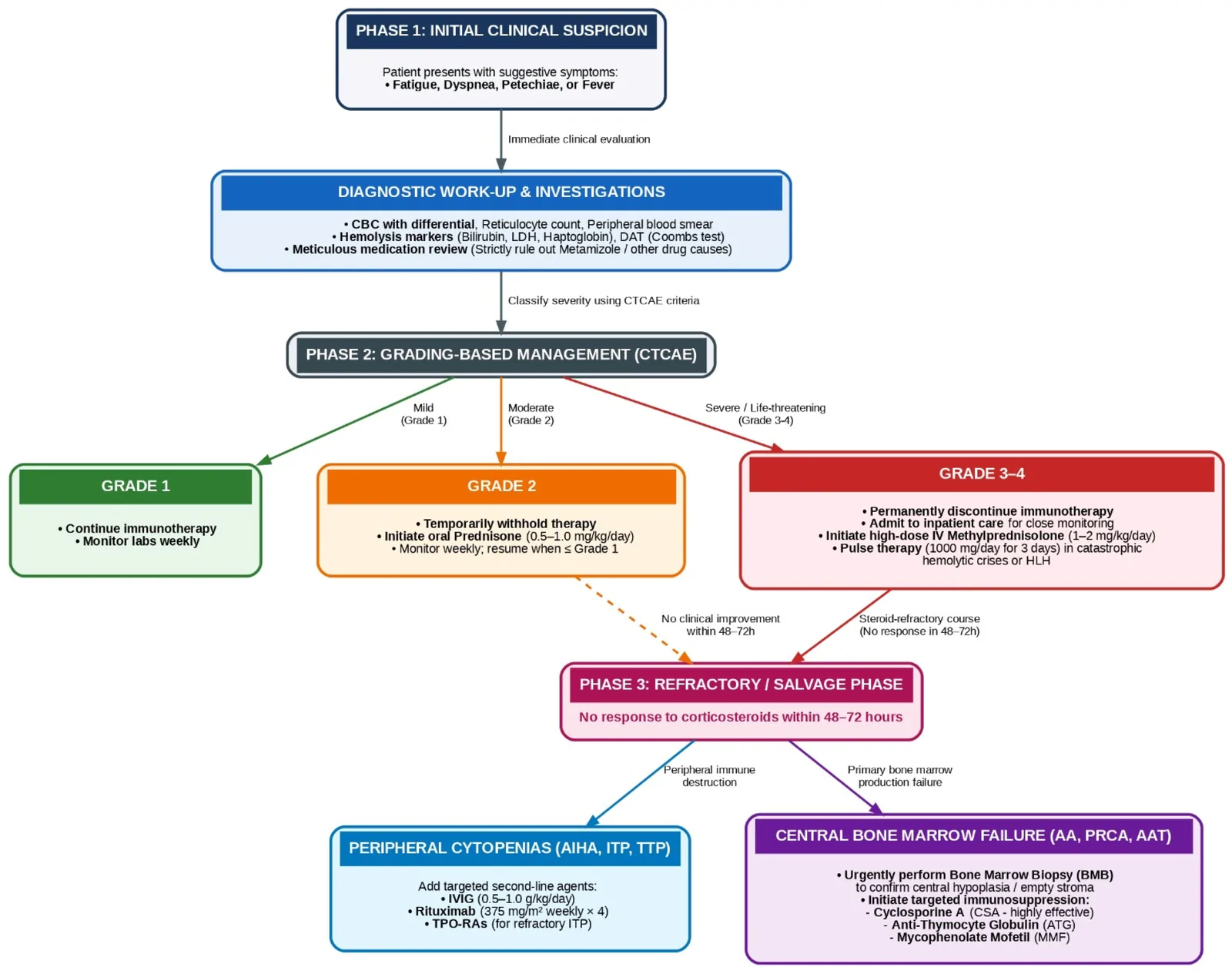
Multi-disciplinary management algorithm for the diagnosis and grading-based treatment of immune-related hematological adverse events (Hem-irAEs).

Alongside immunosuppression, the absolute necessity of acute supportive care measures cannot be overstated. Supportive interventions, such as red blood cell and platelet transfusions, are essential to quickly stabilize patients presenting with highly symptomatic anemia or life-threatening thrombocytopenia while the immunosuppressive agents take effect [33,45,99,113]. When integrating TILs and pembrolizumab, utilizing a lower-dose IL-2 regimen may help mitigate the rates of severe febrile neutropenia and reduce hospitalization times without compromising clinical efficacy [7]. While standardized grading systems guide intervention, distinguishing between moderate and severe toxicity grades can sometimes be subjective, making individual clinical judgment essential in optimizing immunosuppressive therapy [76] (see Figure 2).

Prompt dose reductions or intermittent treatment schedules are standard and highly effective strategies for managing targeted therapy–induced bone marrow suppression typically preventing clinically significant bleeding events and enabling rapid recovery of platelet and neutrophil counts through supportive care while, in the case of immune-related toxicities including rare hematological manifestations, the prompt initiation of systemic corticosteroids remains the first-line intervention and does not appear to negatively impact OS or time to treatment failure [32,50,53].

#### 6.2.2. Lineage-Specific First-Line Adjuncts: The Role of G-CSF in Neutropenia

For patients who develop ir-neutropenia, the administration of G-CSF is a critical, guideline-directed adjunct to rapidly restore neutrophil counts and reduce the duration of severe (grade 4) neutropenia [17,35]. According to current consensus and clinical practice, G-CSF should be administered daily until the leukocyte or absolute neutrophil count safely recovers [35,36]. Retrospective multicenter analyses demonstrate that the vast majority of patients with high-grade ir-neutropenia respond effectively to G-CSF therapy, making it an indispensable first-line intervention alongside the permanent or temporary discontinuation of the offending ICI [15,36]. In cases where profound neutropenia or febrile neutropenia arises from combining novel agents with PD-1 inhibitors, prompt administration of G-CSF typically facilitates rapid recovery [96].

The management of immune-induced neutropenia presents a unique therapeutic challenge because standard high-dose corticosteroids may paradoxically increase the risk of systemic bacterial and fungal infections, potentially acting counterproductively and promoting life-threatening sepsis [15,36]. Consequently, some guidelines and experts advise against the systematic administration of corticosteroids in this specific context without firm evidence of their necessity, prioritizing the early use of G-CSF instead to mitigate infection risks [35,36]. Furthermore, the immediate initiation of empiric broad-spectrum intravenous antibiotics is absolutely mandatory for any patient presenting with febrile neutropenia [35,36]. In cases of prolonged neutropenia, persistent fever, or suspected secondary complications, the rapid addition of empiric antimycotic therapies (and occasionally antivirals) must also be strongly considered to prevent fatal infectious outcomes [15,36]. In clinical scenarios where iAErs demonstrate marked resistance to initial high-dose corticosteroid therapy, the targeted administration of TNF-alpha inhibitors such as infliximab has proven highly efficacious in rescuing patients [120]. For novel targeted combinations that induce high rates of febrile neutropenia, prompt dose reduction in the concurrent chemotherapeutic agent and proactive management with growth factors are critical [89].

#### 6.2.3. Management of Steroid-Refractory Peripheral Cytopenias (ITP and AIHA)

For patients presenting with ITP and AIHA who fail to respond adequately to initial high-dose corticosteroids, or in cases of life-threatening bleeding or profound anemia, IVIG serves as a highly effective early second-line or concomitant therapy. Data syntheses demonstrate that IVIG rapidly counteracts peripheral immune destruction, inducing a prompt rise in platelets or hemoglobin [34,35,112]. Clinical guidelines typically recommend administering IVIG at a dose of 0.5 to 1 g/kg/day for up to 5 consecutive days to stabilize the patient while long-term immunosuppression takes effect [17,35].

In cases of autoantibody-driven peripheral destruction that prove to be intrinsically steroid-refractory, Rituximab (anti-CD20 therapy) is a targeted and highly successful intervention. As a monoclonal antibody that actively depletes B-cells, rituximab halts the production of the aberrant autoantibodies driving the cytopenia [33,45]. It is recommended as a standard second-line agent for severe, persistent ITP and warm autoimmune hemolytic anemia (wAIHA) [17,34,117]. Furthermore, in cases of cold agglutinin autoimmune hemolytic anemia (cAIHA) a condition where corticosteroids are notoriously ineffective, rituximab is frequently prioritized as the optimal upfront therapeutic choice [35,57].

Beyond direct immunosuppression, TPO-RAs provide an additional, highly effective therapeutic pathway by actively stimulating hematopoiesis. Agents such as eltrombopag or romiplostim are successfully utilized for patients with persistent, refractory ITP who do not achieve remission with steroids or IVIG [34,113]. Moreover, emerging evidence highlights that TPO-RAs are particularly beneficial for reversing immunotherapy-induced AAT, serving as a crucial intervention to rapidly restore megakaryocyte function and stimulate platelet production in the bone marrow [41].

#### 6.2.4. Targeted Immunosuppression for Central Marrow Failure (PRCA, AA, AAT)

Because these central bone marrow failure syndromes (PRCA, AAT, and AA) are fundamentally driven by T-cell cytotoxicity rather than autoantibodies, they are frequently refractory to standard high-dose corticosteroid regimens, necessitating a different therapeutic approach [41,67,71,110]. In such steroid-refractory cases, Cyclosporine A serves as a highly effective, mechanism-based therapeutic alternative. As a calcineurin inhibitor that directly targets and suppresses T-cell activation, Cyclosporine A disrupts the cytotoxic attack on the marrow, consistently leading to disease resolution and the stable recovery of circulating blood cells in these specific immune-mediated conditions [41,71,110]. In cases of severe, ipilimumab-induced autoimmune pancytopenia that prove entirely resistant to high-dose oral corticosteroids, the prompt administration of IVIG is often necessary to successfully restore multilineage hematopoiesis [38].

In more severe, life-threatening AA cases or prolonged, persistent PRCA where patients remain heavily transfusion-dependent despite initial immunosuppression, escalation to even more potent T-cell depleting therapies may be required [17,35]. Guidelines and clinical case data suggest that ATG sometimes utilized alongside cyclosporine can act as a critical salvage therapy to actively deplete the autoreactive T-cell clones, rescue bone marrow function, and prevent fatal outcomes associated with irreversible hematopoietic failure [17,35,73]. Because some liposomal chemotherapies spare the bone marrow elements, they represent a potentially safer alternative for combination regimens in patients unable to tolerate profound peripheral blood cytopenias [51].

#### 6.2.5. Aggressive Protocols for Systemic and Life-Threatening Syndromes (HLH, TTP)

For fulminant hyperinflammatory states such as secondary HLH and severe CRS, standard high-dose corticosteroid therapy alone is frequently insufficient to halt the rapidly progressing cytokine storm [35,54,55]. In these catastrophic scenarios, data syntheses and expert guidelines demonstrate that the early introduction of anti-cytokine therapies, specifically the IL-6 receptor antagonist tocilizumab, effectively breaks the inflammatory cascade and serves as a critical, life-saving intervention [30,35,78]. Utilizing tocilizumab in conjunction with steroids has been shown to yield rapid clinical improvement and normalization of proinflammatory cytokine levels in patients experiencing ICI-associated HLH and severe CRS [30,55].

For patients with rapidly deteriorating or treatment-refractory HLH (e.g., those showing insufficient response after 48 h of initial steroid and anti-IL-6 therapy), the cytotoxic agent etoposide is strongly recommended [35,55]. Etoposide acts as a targeted and potent salvage therapy designed to actively ablate and deplete the hyperactivated macrophages and cytotoxic T-cells driving the underlying immune dysregulation, thereby suppressing the massive and lethal production of inflammatory cytokines [30,94].

Conversely, immune-induced TTP represents an entirely unique medical emergency characterized by the autoantibody-mediated depletion of the ADAMTS-13 enzyme, leading to systemic microvascular thrombosis [83,116]. In the setting of TTP, standard high-dose steroids and IVIG are fundamentally inadequate [115]. Immediate TPE (plasmapheresis) is the absolute cornerstone of treatment, acting urgently to both physically remove the circulating ADAMTS-13 autoantibodies and replenish the deficient enzyme via fresh frozen plasma [83,116]. To achieve sustained remission and target the autoantibody-producing B-cells, plasmapheresis must often be combined with targeted therapies such as rituximab (anti-CD20) or caplacizumab, a recently approved anti-vWF nanobody that prevents platelet aggregation [30,115].

### 6.3. Rechallenge Considerations

#### 6.3.1. The Controversy and High Risk of Recurrence

The clinical decision to re-initiate immunotherapy after a severe hem-irAE remains highly controversial and represents a significant management dilemma. Retrospective analyses and broad pharmacovigilance data consistently demonstrate a relatively high rate of irAE recurrence upon re-exposure to ICIs. Across various studies evaluating patients who were rechallenged after an initial hematological toxicity, the recurrence rates are generally reported to be between 14% and 43%, underscoring the need for extreme caution and a careful evaluation of the risk-to-benefit ratio before resuming therapy [17,30,33].

The risks associated with ICI rechallenge become even more pronounced when considering specific, high-fatality hematological conditions. For immune-mediated cytopenias like AIHA, the recurrence rate following re-administration is notably steep. Current literature and extensive case series suggest that AIHA recurrence upon rechallenge can reach approximately 50% to 77.8% [45,57,97,101]. Given the life-threatening nature and high mortality risk associated with severe hemolysis, rechallenging patients who have previously experienced AIHA is highly questionable and requires intensive multidisciplinary deliberation. Due to the profound memory of the humoral immune response, patients who experience ICI-induced warm AIHA face a substantial and perilous risk of hemolytic recurrence if checkpoint blockade is casually reinitiated [97]. Attempts to rechallenge patients with the same PD-1 inhibitor following an initial episode of severe AIHA frequently result in a rapid and dangerous relapse of hemolysis [101].

Furthermore, the risks of re-exposure extend beyond the simple relapse of the initial toxicity. Patients are not only at risk of experiencing the exact same hem-irAE, but they can also develop completely new (*de novo*) toxicities affecting different organ systems or distinct hematopoietic lineages upon ICI rechallenge [23]. For example, complex clinical scenarios have been documented where patients who were successfully treated for one severe central toxicity, such as PRCA, subsequently developed an entirely different peripheral toxicity, such as acute ITP, upon single-agent immunotherapy rechallenge [44]. This unpredictability highlights the profound and sustained immune dysregulation induced by ICIs and emphasizes the absolute necessity for vigilant, broad-spectrum monitoring if a rechallenge is ultimately pursued.

#### 6.3.2. Strategic Class Switching and De-Escalation

When rechallenge is considered after a severe hematological toxicity, strategic class switching represents a potential approach to bypass the specific immunological trigger. This strategy takes advantage of the distinct mechanisms of action between different checkpoint inhibitor classes. For instance, transitioning a melanoma patient from an anti-PD-1/PD-L1 agent (which had caused severe, grade 4 AIHA) to an anti-CTLA-4 agent (ipilimumab) has been successfully attempted [98]. Following the remission of AIHA with corticosteroids, the introduction of an alternative class of ICI did not trigger a recurrence of the hemolysis, suggesting that changing the therapeutic target can safely sustain antitumor efficacy while avoiding the specific prior toxicity [98].

Another clinical strategy involves de-escalating from combination immunotherapy to monotherapy. In patients who develop severe toxicities while receiving dual checkpoint blockade (e.g., ipilimumab plus nivolumab), permanently discontinuing the combination and rechallenging with anti-PD-1 monotherapy alone is a viable option [23,129]. Retrospective analyses indicate that this approach is generally well-tolerated, with the original toxicity recurring in only a minority, approximately 18% to 20%, of patients upon the reinitiation of the single-agent anti-PD-1 therapy [23,129].

However, clinicians must be cautioned that while switching classes or de-escalating to monotherapy can substantially reduce the risk of toxicity recurrence, it does not entirely eliminate it. Severe and even fatal relapses have still been documented with these approaches [23]. For example, grade 5 (fatal) events have occurred following an anti-PD-1 rechallenge in patients who initially experienced toxicity on combination therapy, highlighting the unpredictable nature of immune priming induced by the initial checkpoint blockade [23]. Therefore, the decision to rechallenge must always be carefully individualized, weighing the risk of severe relapse against the available alternative treatment options.

#### 6.3.3. Clinical Decision-Making and Patient Selection

Current clinical guidelines strongly advise against rechallenge and instead recommend permanent ICI discontinuation in several high-risk clinical scenarios. Rechallenge is generally contraindicated in cases of life-threatening (grade 4) hematological irAEs, events that proved to be intrinsically steroid-refractory, or cases requiring the use of prolonged or second-line immunosuppressants to achieve resolution [17,120,129]. Furthermore, permanent discontinuation is recommended if the patient has already achieved a complete or near-complete tumor response, as the oncological benefit of resuming therapy in these cases is outweighed by the severe risk of toxicity recurrence [17,129].

When rechallenge is considered a viable clinical option, strict prerequisites must be met before re-initiating therapy. Re-exposure should only be attempted after the initial toxicity has sufficiently resolved to Grade 1 or back to the patient’s baseline [17]. Additionally, the patient must have successfully tapered off all acute immunosuppression or be safely controlled on a minimal maintenance dose of ≤10 mg/day of prednisone equivalents prior to resuming the offending agent [17].

Ultimately, the decision to rechallenge must be highly individualized, requiring a careful calculation of the risk-benefit ratio for each specific patient. Clinicians may lean toward attempting a rechallenge only if the initial toxicity was relatively mild, or if the adverse event occurred very early in the treatment course, strongly suggesting that the maximum oncological benefit has not yet been reached [129]. This strategy is particularly relevant when the patient lacks alternative, life-saving systemic therapies for their advanced melanoma, necessitating a careful, multidisciplinary balance between the threat of a relapsing hematological irAE and the critical need for ongoing disease control [45,129].

### 6.4. Prognostic Implications (The “Double-Edged Sword”)

#### 6.4.1. The Positive Correlation: Low-Grade irAEs as Biomarkers of Efficacy

The administration of ICIs has revealed an established, albeit complex, correlation between the development of irAEs and improved clinical outcomes. Multiple systematic reviews, meta-analyses, and large real-world studies consistently demonstrate that patients who experience irAEs often exhibit significantly higher objective response rates (ORR) and disease control rates than those who do not develop any toxicities [64]. Furthermore, the occurrence of irAEs is strongly translated into survival benefits, with affected patients frequently demonstrating enhanced PFS and prolonged OS [64,108,130]. This phenomenon suggests that the collateral damage to healthy tissues reflects a highly invigorated immune system that is simultaneously mounting a powerful and effective attack against tumor antigens.

Interestingly, this positive prognostic association is not uniform across all toxicities but is most pronounced in patients developing low-grade (grade 1–2) irAEs [108]. Specifically, dermatological toxicities such as vitiligo-like skin reactions and hypopigmentation and endocrine dysfunctions (e.g., thyroiditis) exhibit a particularly strong correlation with improved tumor response and survival metrics [26,108,130]. Furthermore, clinical data indicate that patients experiencing multiple low-grade, multi-system irAEs often show a significantly higher ORR and an even more favorable overall prognosis than those experiencing only a single irAE [64,108]. Together, these findings suggest that these specific, manageable side effects serve as valuable clinical surrogates for a robust, system-wide anti-tumor immune response, acting as tangible biomarkers of therapeutic efficacy [108].

In highly selected scenarios where pancytopenia has resolved with steroid therapy and G-CSF support, continuing anti-PD-1 treatment may be considered if the patient exhibits a robust ongoing anti-tumor response [103]. Deep and durable clinical responses to PD-1 blockade can be achieved even in patients with difficult-to-treat malignancies lacking alternative therapies, further emphasizing the potent immune restoration triggered by these agents [28]. For melanoma patients who achieve a robust objective clinical response to ICI therapy and maintain stable radiological findings, offering a structured ‘drug holiday’ has emerged as a practical strategy to mitigate long-term cumulative toxicities while monitoring for relapse [117]. While PD-L1 expression and tumor mutational burden are frequently investigated as predictive markers, they do not always correlate significantly with recurrence-free survival improvements in melanoma patients receiving adjuvant immunotherapy [4].

#### 6.4.2. The Negative Impact: High-Grade and Hematological Toxicities

In stark contrast to the positive predictive value frequently observed with mild or low-grade irAEs, the onset of high-grade (grade 3–4) and potentially fatal (grade 5) toxicities does not confer a survival advantage. Severe, life-threatening manifestations such as profound hematological toxicities, fulminant myocarditis, severe pneumonitis, and hepatitis are generally not associated with improved long-term clinical outcomes [26,122]. In fact, comprehensive multivariate analyses have demonstrated that while overall irAEs occurrence might sometimes show a positive trend, the specific development of ≥ grade 3 toxicities frequently remains a significant, independent confounder for diminished OS [64,65,130].

The relationship between severe toxicity and efficacy presents a complex clinical paradox. While the development of severe irAEs might sometimes correlate with a robust, immediate anti-tumor immune activation occasionally yielding an improved initial ORR, the ultimate OS benefit is frequently negated [64]. This diminished long-term OS is fundamentally driven by a substantial increase in early, non-cancer-related mortality resulting directly from the life-threatening and destructive nature of the toxicities themselves [122]. Furthermore, the obligatory permanent discontinuation of potentially life-saving immunotherapy and the prolonged requirement for systemic high-dose corticosteroids to manage these severe events can further attenuate the durable anti-tumor efficacy, ultimately worsening the patient’s prognosis [122]. Severe lymphopenia not only predisposes patients to opportunistic infections but also frequently correlates with a diminished response to immune checkpoint blockade and worse OS [48]. The underlying genetic architecture, including the cumulative presence of unfavorable genotypes in DNA repair pathways, significantly modulates melanoma-specific survival and may influence how patients respond to and recover from systemic therapeutic stresses, while specific genetic alterations in cell-cycle regulators can also predict enhanced clinical responses to targeted combinations, highlighting the role of genomic profiling in anticipating both therapeutic efficacy and tolerability in melanoma cohorts [65,86].

#### 6.4.3. Confounding Factors: Immunosuppression and Treatment Interruption

The interpretation of the efficacy-toxicity relationship in severe irAEs is significantly confounded by necessary clinical interventions, primarily early treatment cessation. The emergence of high-grade and life-threatening hematological irAEs usually mandates the immediate and permanent discontinuation of the offending ICI [17,56]. Consequently, this abrupt cessation prevents the patient from receiving the full therapeutic benefit of the intended treatment course, which inherently limits ongoing disease control and ultimately attenuates the potential for a durable anti-tumor response [122].

Furthermore, the clinical management of severe irAEs presents a paradoxical challenge that further confounds prognostic outcomes. Because hematological and other severe irAEs represent a state of profound systemic immune hyperactivation, they require aggressive intervention with high-dose systemic corticosteroids, and occasionally, secondary immunosuppressive agents to mitigate life-threatening tissue damage [26,119]. While essential for patient survival, this profound iatrogenic immunosuppression particularly when administered early in the treatment course or at high doses can inadvertently attenuate the ICI-induced anti-tumor immune response [121,122]. Mechanistically, prolonged or high-dose corticosteroids can promote T regulatory cell expansion, induce apoptosis in lymphocytes, and blunt the proliferative burst of effector CD8+ T-cells required for tumor destruction, thereby contributing to disease progression and poorer OS outcomes in these vulnerable patient cohorts [119,121].

## 7. Conclusions

The advent of ICIs and targeted therapies has fundamentally redefined the prognostic landscape of advanced melanoma, shifting the paradigm from invariably poor outcomes to significant prolongations in OS. However, this unprecedented therapeutic efficacy has introduced a distinct and often unpredictable spectrum of irAEs. Among these, Hem-irAEs emerge as a uniquely perilous subset; despite their overall rarity, they carry a disproportionately high mortality burden, particularly in cases of catastrophic immune-mediated AA, which are often refractory to standard-of-care treatments.

The intricate pathophysiology of these events spanning autoantibody-driven peripheral destruction (such as AIHA and ITP), T-cell-mediated central marrow failure (including PRCA and acquired amegakaryocytic thrombocytopenia), and explosive systemic cytokine storms like HLH underscores the urgent need for meticulous, tiered diagnostic protocols. Distinguishing true immune-mediated cytopenias from disease progression or idiosyncratic drug reactions (such as metamizole-induced agranulocytosis) requires heightened clinical vigilance, strict baseline monitoring, and the critical early utilization of bone marrow biopsies to differentiate peripheral consumption from central hematopoietic collapse.

Navigating these toxicities presents oncologists with a profound clinical paradox, accurately characterized as a “double-edged sword.” While the emergence of low-grade irAEs frequently acts as a positive prognostic surrogate for a robust anti-tumor response and enhanced survival, high-grade Hem-irAEs negate this oncological benefit by driving massive increases in non-cancer-related mortality. Furthermore, the obligatory administration of early high-dose corticosteroids and targeted secondary immunosuppressants (e.g., rituximab, tocilizumab, or cyclosporine) to rescue patients from fulminant failure intrinsically risks blunting the very anti-tumor immune responses the therapies were designed to unleash.

Moving forward, the future of melanoma therapy must prioritize proactive risk stratification to mitigate these fatal events. The routine integration of predictive biomarkers including baseline autoantibody screening, genetic profiling of HLA susceptibility loci, and dynamic systemic inflammatory indices (like the NLR) holds immense potential to identify highly vulnerable patient populations prior to treatment. Ultimately, as combination immunotherapies continue to amplify toxicity risks, optimizing patient outcomes will demand a highly personalized, multidisciplinary approach that carefully balances the life-saving potential of checkpoint blockade against the catastrophic threat of hematopoietic failure, particularly when navigating the high-risk and controversial dilemmas of treatment rechallenge.

## Figures and Tables

**Table 1:** Methodological and statistical analysis types employed in the included immunotherapy literature.

Study Design Models	Data Analysis Methods	Primary Goal and Usage in Hem-irAE Research	Example Refs
Large-scale pharmacovigilance databases (e.g., FAERS, VigiBase)	Disproportionality analysis, ROR with 95% confidence intervals, IC, PRR	Detect safety signals and assess risk patterns of rare, life-threatening hematological adverse events across massive, real-world patient populations	[42,58,59]
Systematic reviews and meta-analyses of clinical trials	Fixed-effects and random-effects models, Cochran Q test, I-squared (I2) index test for heterogeneity, RR calculations, Labbe plots, publication bias tests	Aggregate the overall and high-grade incidence rates of hematological toxicities across multiple RCTs and perform direct statistical comparisons with chemotherapy	[9,60,61,62,63]
Prospective and retrospective cohort studies	Kaplan–Meier survival curves, Log-rank tests, univariable and multivariable Cox proportional hazards regression models for HR estimations	Evaluate patient-level outcomes, including OS and PFS; identify prognostic risk factors and genetic associations	[24,64,65]
Case reports and retrospective case series	Descriptive statistics (medians, ranges, counts, and percentages), Pierson-5 quality scoring index, Naranjo probability scale for adverse drug reaction causality	Provide clinical description of patient-level onset kinetics, CBC curves, bone marrow biopsy findings, steroid tapering timelines, and salvage rescue responses	[45,56,66,67]

**Abbreviations:** CBC, complete blood count; FAERS, FDA Adverse Event Reporting System; HR, hazard ratio; IC, information component; OS, overall survival; PFS, progression-free survival; PRR, proportional reporting ratio; RCTs, randomized controlled trials; ROR, reporting odds ratio; RR, risk ratio.

**Table 2:** Foundational patient cohorts and landmark studies evaluating hematological toxicities under checkpoint blockade.

Study	Study Design and Phase	Patient Count (*n*)	Major Cancer/Tumor Types	Primary Hematological Findings
Nishijima et al., 2017 [60]	Systematic meta-analysis of 10 phase II/III randomized controlled trials	*n* = 5744 (2912 on ICIs; 2832 on Chemotherapy)	Advanced melanoma, NSCLC, RCC	Demonstrated a significantly lower incidence of treatment-related all-grade neutropenia (0.5% vs. 16.1%) and thrombocytopenia (0.6% vs. 7.0%) with PD-1/PD-L1 inhibitors compared with chemotherapy.
Delanoy et al., 2019 [33]	Retrospective nationwide registry (French RECAM registry)	*n* = 35 confirmed severe hematological irAE cases	Metastatic melanoma, NSCLC, lymphomas	ITP (29%) and wAIHA, (16%) as the most prevalent cytopenias, with a median onset of 10 weeks and poor resolution in AA.
Boegeholz et al., 2020 [15]	Retrospective multicenter case series and pooled review	*n* = 34 patients with severe, biopsy-confirmed isolated neutropenia	Metastatic melanoma, NSCLC, uveal melanoma	Reported a median onset of 10.5 weeks. Neutropenia was deep and life-threatening but achieved 91% complete recovery with a combination of daily G-CSF and systemic corticosteroids.
Yan et al., 2025 [64]	Real-world retrospective observational cohort study	*n* = 2523 patients receiving immunotherapy	HCC, gastric cancer, melanoma, NSCLC	Documented a real-world hem-irAE incidence of ~0.5–1.0% in clinical practice, emphasizing the significantly higher risk of severe multi-lineage cytopenias during anti-PD-1 + anti-CTLA-4 dual combinations.
Bisiou et al., 2026 [42]	Nationwide retrospective cohort and pharmacovigilance (VigiBase) analysis	*n* = 9 registry cases + 185 VigiBase cases	Metastatic melanoma, NSCLC, RCC	Characterized immune-related PRCA as a late-onset, highly transfusion-dependent central toxicity, demonstrating excellent response rates (80%) to targeted T-cell salvage with Cyclosporine A.

**Abbreviations:** AA, aplastic anemia; CTLA-4, cytotoxic T-lymphocyte–associated protein 4; G-CSF, granulocyte colony-stimulating factor; HCC, hepatocellular carcinoma; hem-irAE, hematological immune-related adverse event; ICI, immune checkpoint inhibitor; ITP, immune thrombocytopenia; *n*, sample size; NSCLC, non-small cell lung cancer; PD-1, programmed cell death protein 1; PD-L1, programmed death-ligand 1; PRCA, pure red cell aplasia; RCC, renal cell carcinoma; wAIHA, warm autoimmune hemolytic anemia.

**Table 3:** Characteristics, incidence, and risk profiles of hematological irAEs across different classes of checkpoint inhibitors and combination regimens.

Therapeutic Strategy	Representative Agents	All-Grade Incidence (%)	Severe (Grades 3–4) Incidence (%)	Predominant Clinical Spectrum	Refs
Anti-CTLA-4 Monotherapy	Ipilimumab Tremelimumab	~0.5–1.0%	~0.1–0.3%	Neutropenia, thrombocytopenia, mild anemia; rare severe AA.	[56,75,76]
Anti-PD-1/PD-L1 Monotherapy	Nivolumab, Pembrolizumab, Atezolizumab, Cemiplimab, Avelumab, Durvalumab	~3.6–4.7%	~0.5–0.7%	ITP, AIHA, PRCA.	[33,60,61,62]
Dual Checkpoint Blockade	Ipilimumab + Nivolumab (or Durvalumab + Tremelimumab)	~6.0–10.0%	~1.5–2.5%	Severe multi-lineage cytopenias (bicytopenia, pancytopenia), AA, HLH, and TTP.	[24,25,56,83]
Targeted/Cytotoxic Combination	MEK/BRAF Inhibitors + Chemo (or Anti-PD-1 + Nab-Paclitaxel)	Highly variable (up to 30–40%)	~10.0–20.0%	Overlapping treatment-induced myelosuppression, severe neutropenia, and delayed platelet recovery.	[5,84,85,86]

**Abbreviations:** AA, aplastic anemia; AIHA, autoimmune hemolytic anemia; BRAF, B-Raf proto-oncogene serine/threonine kinase; CTLA-4, cytotoxic T-lymphocyte–associated protein 4; HLH, hemophagocytic lymphohistiocytosis; ITP, immune thrombocytopenia; MEK, mitogen-activated protein kinase kinase; PD-1, programmed cell death protein 1; PD-L1, programmed death-ligand 1; PRCA, pure red cell aplasia; TTP, thrombotic thrombocytopenic purpura.

## Data Availability

Data is contained within the article.

## Abbreviations

AA	Aplastic anemia
AAT	Acquired amegakaryocytic thrombocytopenia
ADs	Autoimmune diseases
AIHA	Autoimmune hemolytic anemia
ANA	Antinuclear antibodies
aPTT	Activated partial thromboplastin time
ATG	Anti-thymocyte globulin
BMB	Bone marrow biopsy
BMI	Body mass index
cAIHA	Cold agglutinin autoimmune hemolytic anemia
CBC	Complete blood count
CLL	Chronic lymphocytic leukemia
CRS	Cytokine release syndrome
CTLA-4	Cytotoxic t-lymphocyte-associated protein 4
DAT	Direct antiglobulin test (coombs test)
DIC	Disseminated intravascular coagulation
DLTs	Dose-limiting toxicities
EBV	Epstein–Barr virus
G-CSF	Granulocyte colony-stimulating factor
GM-CSF	Granulocyte-macrophage colony-stimulating factor
Hem-irAEs	Hematological immune-related adverse events
HLA	Human leukocyte antigens
HLH	Hemophagocytic lymphohistiocytosis
ICI/ICIs	Immune checkpoint inhibitor/inhibitors
IFN-γ	Interferon-gamma
IL-1β	Interleukin-1β
IL-2	Interleukin-2
IL-6	Interleukin-6
IL-17	Interleukin-17
irAEs	Immune-related adverse events
ir-neutropenia	Immune-related neutropenia
ir-TCP	Immune-related thrombocytopenia
ITP	Immune thrombocytopenia/idiopathic thrombocytopenic purpura
IVIG	Intravenous immunoglobulin
LDH	Lactate dehydrogenase
LGL	Large granular lymphocytosis
MAHA	Microangiopathic hemolytic anemia
NLR	Neutrophil-to-lymphocyte ratio
ORR	Objective response rates
OS	Overall survival
PD-1	Programmed cell death protein 1
PD-L1	Programmed cell death protein 1 ligand
PFS	Progression-free survival
PH	Paraneoplastic hyperleucocytosis
PRCA	Pure red cell aplasia
sCD25	Soluble il-2 receptor
SNPs	Single nucleotide polymorphisms
TPE	Therapeutic plasma exchange
TPO-RAs	Thrombopoietin receptor agonists
Treg	Regulatory T cell
TTP	Thrombotic thrombocytopenic purpura
vWF	Von Willebrand factor
wAIHA	Warm autoimmune hemolytic anemia
